# A meta-analysis showing improved cognitive performance in healthy young adults with transcranial alternating current stimulation

**DOI:** 10.1038/s41539-022-00152-9

**Published:** 2023-01-03

**Authors:** Tae Lee Lee, Hanall Lee, Nyeonju Kang

**Affiliations:** 1 Department of Human Movement Science, Incheon National University, Incheon, South Korea; 2 Neuromechanical Rehabilitation Research Laboratory, Incheon National University, Incheon, South Korea; 3 Division of Sport Science & Sport Science Institute, Incheon National University, Incheon, South Korea

**Keywords:** Inhibition-excitation balance, Cognitive control, Motor control

## Abstract

Transcranial alternating current stimulation (tACS) is a non-invasive brain stimulation used for improving cognitive functions via delivering weak electrical stimulation with a certain frequency. This systematic review and meta-analysis investigated the effects of tACS protocols on cognitive functions in healthy young adults. We identified 56 qualified studies that compared cognitive functions between tACS and sham control groups, as indicated by cognitive performances and cognition-related reaction time. Moderator variable analyses specified effect size according to (a) timing of tACS, (b) frequency band of simulation, (c) targeted brain region, and (b) cognitive domain, respectively. Random-effects model meta-analysis revealed small positive effects of tACS protocols on cognitive performances. The moderator variable analyses found significant effects for online-tACS with theta frequency band, online-tACS with gamma frequency band, and offline-tACS with theta frequency band. Moreover, cognitive performances were improved in online- and offline-tACS with theta frequency band on either prefrontal and posterior parietal cortical regions, and further both online- and offline-tACS with theta frequency band enhanced executive function. Online-tACS with gamma frequency band on posterior parietal cortex was effective for improving cognitive performances, and the cognitive improvements appeared in executive function and perceptual-motor function. These findings suggested that tACS protocols with specific timing and frequency band may effectively improve cognitive performances.

## Introduction

Cognitive processes are related to exchanging neuronal signals in a specific manner across widely distributed brain regions^1,2^. Given that a large number of cortical and sub-cortical regions are functionally interconnected, altered neural activation patterns in specific brain area simultaneously influence neural activations in other brain region^3,4^. Specifically, the temporal synchronization of rhythmic oscillations across key brain regions may be crucial neurophysiological mechanism for mediating functional neural networks contributing to information processing and communications^5–9^. For example, the signal synchronization across pre-synaptic spikes within sending neuron populations in one or several cortical regions may effectively drive activities of post-synaptic neuronal populations in receiving regions^10–12^. Interestingly, synchronized oscillations of neuronal populations in a certain frequency band may be associated with advanced cognitive functions^13,14^.

Previous studies raised a possibility that neural oscillations at specific frequency band predominantly appears in various cognitive processes^15,16^. For example, increased synchronized oscillations at the theta frequency band (4–7 Hz) may be associated with improved executive function / complex attention and learning and memory^17,18^. Specifically, a classical animal study that used the electrocorticogram reported greater neural oscillations at the theta frequency band in rat hippocampal pyramidal neurons during spatial navigation tasks^19^. Moreover, theta rhythmic neural oscillations were observed in the human prefrontal cortex (PFC) while remembering a list of items^20,21^. Greater neural synchronization in brain at the alpha frequency band (8–12 Hz) may be related to executive function and complex attention^22^. Several electrophysiological studies evidence higher alpha rhythmic neural synchronization across PFC and parietal cortical areas while generating creative ideas^23,24^, and further these oscillation patterns was linked to improved inhibitory functions^25,26^. In addition, greater brain oscillation at the beta frequency band (13–30 Hz) presumably improved the executive function / complex attention^27,28^. Specifically, beta frequency power in PFC and primary motor cortex (M1) increased during preparatory and inhibitory phases for the movement execution, whereas beta frequency power decreased after the movement execution^29–31^. Presumably, neural oscillation patterns at the gamma frequency band (31–139 Hz) influenced the executive function, complex attention, and social cognition^16^. Gamma waves emerge in the animal parietal and frontal regions during attentive behavioral states such as a cat observing prey in a room^32^, and further were activated while integrating sensory information^33,34^. Taken together, modulating the synchronization of brain oscillations at a specific frequency band may effectively facilitate improvement in various cognitive functions.

Transcranial alternating current stimulation (tACS), one of the non-invasive brain stimulation technique, has been developed to modulate brain oscillations at certain frequency band for enhancing either cognitive or motor functions^35–37^. tACS protocols use weak sinusoidal oscillating electrical currents into the scalp to temporarily synchronize the neural firing timing^38,39^. Thus, the rhythmically reversed electron flow potentially interacts with endogenous oscillations in the brain^40,41^ as previous electroencephalogram (EEG) studies suggested entrained endogenous brain oscillations and external currents^37,42,43^. Interestingly, recent literature review studies raised a possibility of positive effects of tACS protocols on cognitive functions^38,44^. Moreover, some prior studies suggested that timing of tACS protocols (e.g., tACS protocols during cognitive tasks: online stimulation and tACS protocols before cognitive tasks: offline stimulation) may induce different effects on cognitive functions^45,46^. For example, online-tACS protocols may facilitate higher entrainment between ongoing neural oscillation and external electrical oscillations^40^, whereas offline-tACS protocols may cause longer lasting after-effects presumably contributing to network changes related to neural plasticity^47^. Thus, determining potential treatment effects of tACS interventions based on different stimulation timing can provide meaningful information on identifying optimal stimulation protocols facilitating cognitive functions.

The purpose of this systematic review and meta-analysis was to investigate the effect of tACS protocols on cognitive functions in healthy young adults. Previous studies suggested that the existence of speed and accuracy trade-off in cognitive processes that hasty responses are error-prone whereas careful decisions take more time^48,49^. Further, different brain involvements were observed between cognitive functions estimated by speed and accuracy, respectively^50^. Thus, we focused on two types of cognitive function variables including cognitive performance and cognition-related reaction time to examine potential altered cognitive functions between active tACS protocols and sham stimulation. In addition, we compared potential different effects of timing of tACS protocols (i.e., online versus offline stimulation) on cognitive function, and further determined whether specific frequency bands for tACS protocols (i.e., delta vs. theta vs. alpha vs. beta vs. gamma vs. ripple) alter cognitive function improvements^40,45^. For each frequency band of online- and offline-tACS protocols, we additionally examined specific treatment effects on cognitive functions based on different targeted brain regions and cognitive domains, respectively^51^.

## Results

### Study identification

**Fig. 1:**
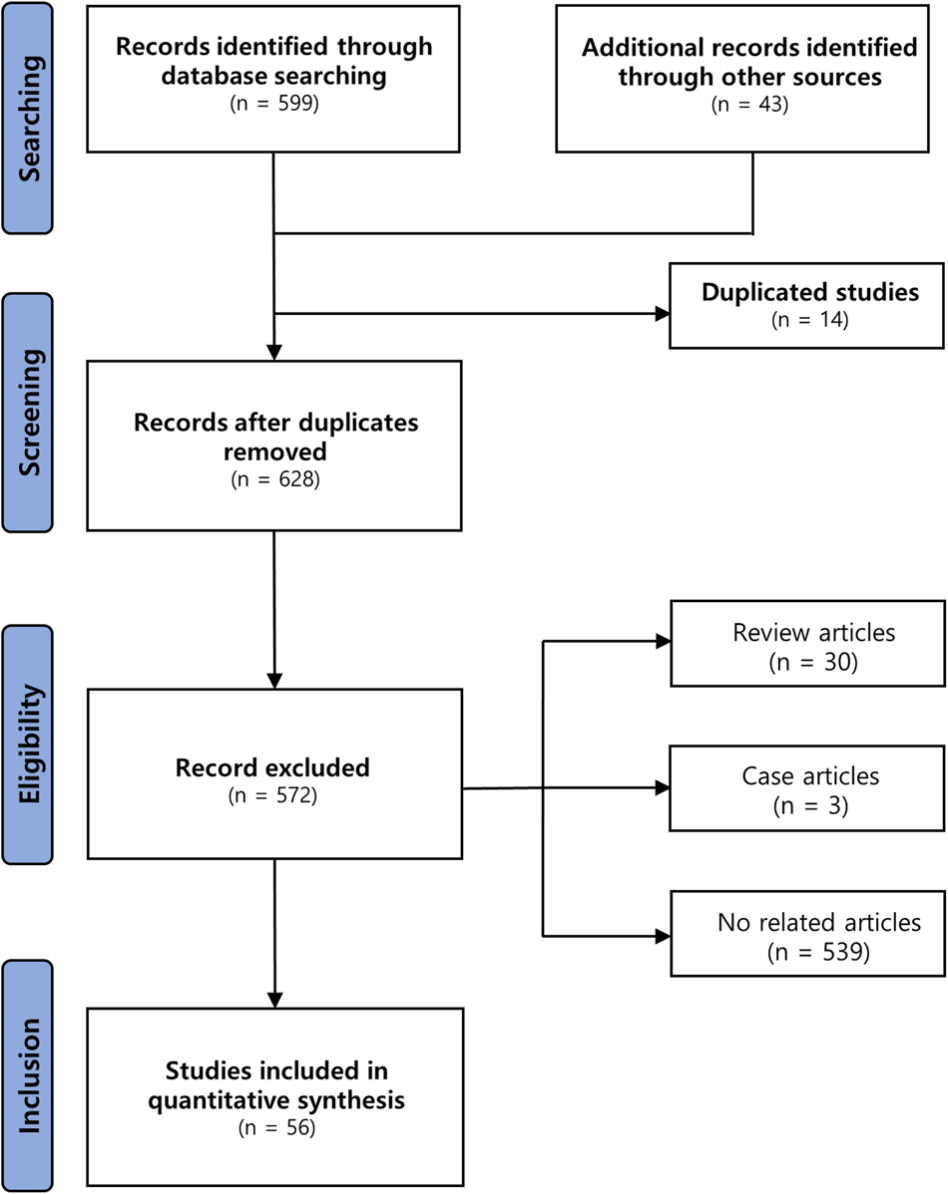
PRISMA flowchart. The flowchart shows the study identification procedure.

Our initial search found 573 potential studies from the PubMed, 26 potential articles from the Web of Science, and 43 articles from other resources, and we removed 14 duplicated articles. In addition, we excluded 572 articles (i.e., 30 review articles, three case articles, and 539 studies irrelevant to our topic). Finally, the remaining 56 studies that examined potential effects of tACS on cognitive functions using either cognitive performance or cognition-related reaction time variables qualified for this meta-analysis^45,46,52–105^. The PRISMA flow diagram illustrating our study identification procedure is shown in Fig. 1.

### Participant characteristics

Fifty-six total qualified studies in this meta-analysis included 1797 healthy young adults without any neurological and psychological deficits (a range of mean age = 18.0–33.0 years and a range of female proportion = 52.8–100%). Nine studies were randomized controlled trials, and 46 studies used a crossover design. One study used both designs for each experiment^65^. Table 1 shows specific detailed demographic information on the participants.

**Table 1:** Demographic information for participants.

Study	Study design	Total *N*	Age (years)	Sex (a ratio of females)
Alekseichuk^52^	Crossover	25	23.5 ± 2.9	13 F 12 M (52.0%)
Alekseichuk^84^	Crossover	25	18–28	13 F 12 M (52.0%)
Ambrus^53^	Crossover	18	24.6 ± 3.2	12 F 6 M (66.7%)
Antonenko^54^	Crossover	12	22.3 ± 1.5	6 F 6 M (50.0%)
Brauer^55^	Crossover	23	22.9 ± 3.4	16 F 7 M (69.6%)
Braun^85^	Crossover	Exp (1) 36 Exp (2) 36	20.0 ± 2.4 21.0 ± 2.2	24 F 12 M (66.7%) 24 F 12 M (66.7%)
Brignani^56^	RCT	96	21.8 ± 2.5	48 F 48 M (50.0%)
Deng^86^	Crossover	Exp (1) 20 Exp (2) 18	21.2 ± 3.0 22.1 ± 2.4	13 F 7 M (65.0%) 15 F 3 M (83.4%)
Feurra^45^	Crossover	14	27.6 ± 4.3	8 F 6 M (57.1%)
Fusco^57^	Crossover	36	24.4 ± 3.5	18 F 18 M (50.0%)
Giustiniani^58^	Crossover	17	24.5 ± 3.5	NR
Grabner^87^	Crossover	22	23.0 ± 2.9	11 F 11 M (50.0%)
Gutteling^88^	Crossover	22	18–31	12 F 10 M (54.5%)
Hopfinger^89^	Crossover	23	18–27	14 F 9 M (60.9%)
Hoy^59^	Crossover	18	29.3 ± 7.7	9 F 9 M (50.0%)
Janik^60^	Crossover	22	25.7 ± 5.8	13 F 9 M (61.9%)
Jaušovec (BP)^61^	Crossover	24	20.7 ± 5.6	16 F 8 M (66.7%)
Jaušovec (AP)^62^	Crossover	36	20.5 ± 4.3	27 F 9 M (75.0%)
Javadi^90^	Crossover	17	22.1 ± 2.7	10 F 7 M (58.8%)
Kasten^91^	RCT	20	26.0 ± 3.0	8 F 12 M (40.0%)
Laczó^92^	Crossover	20	25.8 ± 6.2	9 F 11 M (45.0%)
Lang^63^	RCT	37	26.7 ± 5.8	18 F 19 M (48.6%)
Loffler^64^	RCT	23	25.7 ± 2.7	12 F 11 M (52.2%)
Luft^65^	Crossover RCT	Exp (1) 29 Exp (2) 36	24.6 ± 5.9 23.9 ± 4.5	15 F 14 M (50.0%) NR
Lustenberger^66^	Crossover	Exp (1) 19 Exp (2) 20	20.9 ± 2.7 20.5 ± 3.2	14 F 5 M (73.7%) 7 F 13 M (35.0%)
Marchesotti^67^	Crossover	15	25.6 ± 7.8	11 F 4 M (73.3%)
Meier^68^	Crossover	26	28.5 ± 7.9	8 F 18 M (30.7%)
Meiron^69^	RCT	24	21.5 ± 2.1	24 F (100%)
Meng^70^	Crossover	18	21.7 ± 2.8	12 F 6 M (66.7%)
Moliadze^93^	Crossover	24	22.0 ± 3.4	12 F 12 (50.0%)
Neubauer^94^	Crossover	20	24.9 ± 3.3	11 F 9 M (55.0%)
Nomura^71^	RCT	36	21.3 ± 0.5	28 F 8 M (77.8%)
Pahor^72^	Crossover	28	20.8 ± 4.4	20 F 8 M (71.4%)
Pahor^95^	Crossover	18	20.2 ± 0.4	11 F 7 M (61.1%)
Polanía^73^	Crossover	36	22–30	NR
Polanía^74^	Crossover	86	20–30	30 F 56 M (34.9%)
Pollok^75^	Crossover	13	22.1 ± 2.6	7 F 6 M (53.8 %)
Reinhart^96^	Crossover	Exp (1) 30 Exp (2) 30 Exp (3) 30	26 27 26	14 F 16 M (46.7%) 16 F 14 M (53.3%) 15 F 15 M (50.0%)
Riecke^97^	Crossover	20	20–38	9 F 11 M (45.0%)
Riecke^98^	Crossover	20	20–28	10 F 10 M (50.0%)
Santarnecchi^76^	Crossover	20	20.2 ± 12.3	10 F M 10 (50.0%)
Santarnecchi^77^	Crossover	Exp (1) 24 Exp (2) 34	24.1 ± 3.0	28 F 30 M (48.2%)
Santarnecchi^78^	Crossover	31	24.4 ± 3.8	17 F 14 M (54.8%)
Schuhmann^79^	Crossover	34	21.6±NR	18 F 16 M (51.4%)
Sela^80^	RCT	27	23.9 ± 2.5	14 F 13 M (51.9%)
Strüber^99^	Crossover	Exp (1) 17 Exp (2) 13 Exp (3) 15	24.9 ± 4.1	9 F 8 M (52.9%) 9 F 4 M (69.2%) 9 F 6 M (60.0%)
Tseng^100^	Crossover	Exp (1) 20 Exp (2) 20	21 23	8 F 12 M (40.0%) 8 F 12 M (40.0%)
Tseng^101^	Crossover	Exp (1) 24 Exp (2) 24	23 23	12 F 12 M (50.0%) 12 F 12 M (50.0%)
Violante^81^	Crossover	10	28.6 ± 5.0	6 F 4 M (60.0%)
Vosskuhl^46^	RCT	33	25.8 ± 2.7	14 F 19 M (42.4%)
Wischnewski^82^	RCT	50	24.1 ± 7.8	31 F 19 M 62.0%)
Wöstmann^102^	Crossover	20	19–31	10 F 10 M (50.0%)
Wynn^83^	Crossover	54	21.3 ± 2.7	38 F 16 M (70.3%)
Zavecz^103^	Crossover	26	21.4 ± 1.5	19 F 7 M (73.1%)
Zoefel^104^	Crossover	17	33.0 ± 8.0	10 F 7 M (58.8%)
Zoefel^105^	Crossover	Exp (1) 27 Exp (2) 19	31.0 ± 7.0 21.0 ± 2.0	15 F 12 M (55.6%) 8 F 11 M (42.1%)

*AP* published in the Acta Psychologica, *BP* published in the Biological Psychology, *Exp* experiment, *F* female, *M* male, *NR* not reported, *RCT* randomized controlled trial.

Data for age is mean ± standard deviation.

### tACS protocols and potential side effects

For improving cognitive functions, the qualified studies used tACS protocols stimulating regions of (a) prefrontal cortex (PFC) including primary motor cortex, dorsolateral-prefrontal cortex, inferior frontal gyrus, and frontal region = 21 studies, (b) posterior parietal cortex (PPC) including posterior occipital cortex and parietal cortex = 19 studies, (c) temporal cortex (TC) including fusiform cortex and temporal region = seven studies, and (d) multiple regions (Multi) such as targeting multiple regions across PFC, PPC, and TC = eight studies. One study focused on two different regions including PPC and TC, respectively^78^. For the timing of tACS protocols, 38 studies used tACS protocols during cognitive tasks (i.e., online-tACS), and 12 studies applied tACS protocols prior to executing cognitive tasks (i.e., offline-tACS). Six studies examined both timings of tACS protocols, respectively^46,55,58,65,86,96^. Twenty-eight out of 56 total studies administered only tACS protocols, whereas the remaining 28 studies applied tACS protocols with additional task-related trainings (e.g., brief training phase, discrimination task, familiarization session, language assessment, training visual associative memory task, and word-pair learning). Forty-eight studies administered a single session of tACS protocols, whereas eight studies applied multiple sessions of tACS protocols (i.e., 2–4 sessions).

The specific parameters of tACS protocols used for the qualified studies were: (a) stimulation intensity = 0.7–3 mA, (b) electrode area = 1.2–35 cm^2^, (c) current density = 0.02–0.83 mA/cm^2^, (d) density charge = 0.24–16.8 C/cm^2^, and (e) session duration = 2 s–48 min. Specific frequency bands for tACS protocols included: (a) delta band (1–3 Hz) = four studies, (b) theta band (4–7 Hz) = 30 studies, (c) alpha band (8–12 Hz) = 19 studies, (d) beta band (13–30 Hz) = seven studies, (e) gamma band (31–139 Hz) = 24 studies, and (f) ripple band (140 Hz) = one study. Specific details on tACS protocols are shown in Table 2.

**Table 2:** Specific parameters for tACS protocols.

Study	Timing	Anodal	Return	Session	Intensity, area, density, duration, density charge	Frequency band
Alekseichuk^52^	Online	Multi: L-PFC + L-PPC	R-PFC + R-PPC	1	1 mA, 25 cm^2.^, 0.04 mA/cm^2^, 18 min, 0.72 C/cm^2^	Theta (6 Hz)
Alekseichuk^84^	Online	PPC: R-PPC	R-TC + M1+ L-PPC + POC	1	3 mA, 4 cm^2^, 0.75 mA/cm^2^, 20 min, 15.0 C/cm^2^	Theta (4 Hz)
Ambrus^53^	Offline	PFC: Bi-PFC	Bi-Mastoids A	1	1 mA, 25 cm^2^, 0.04 mA/cm^2^, 10 min, 0.40 C/cm^2^	Ripple (140 Hz)
Antonenko^54^	Offline	PPC: L-PPC	R-Supraorbital A	1	1 mA, 35 cm^2^, 0.03 mA/cm^2^, 20 min, 0.60 C/cm^2^	Theta (6 Hz)
Brauer^55^	Online Offline	PFC: R-PFC	L-Supraorbital A	1	1 mA, 25 cm^2^, 0.04 mA/cm^2^, 20 min, 0.80 C/cm^2^	Theta (6 Hz)
Braun^85^	Online	PFC: R-IFG, PFC: L-IFG	L-SO A, R-SO A	1	Exp (1) 2 mA, 14 cm^2^, 0.14 mA/cm^2^, 2 s, 0.28 C/cm^2^ Exp (2) 1.6 mA, 10.75 cm^2^, 0.15 mA/cm^2^, 2 s, 0.30 C/cm^2^	Theta (6.8 Hz), Alpha (10.7 Hz), Beta (18.5 Hz), Gamma (30, 48 Hz)
Brignani^56^	Online	PPC: Bi-POC	Vertex (Cz)	1	1 mA, 16 cm^2^, 0.06 mA/cm^2^, 15 min, 0.90 C/cm^2^	Theta (6 Hz), Alpha (10 Hz), Beta (25 Hz)
Deng^86^	Online Offline	PPC: R-PPC	R-PPC + M-PPC R-PPC + R-POC	1	1.5 mA, 4 cm^2^, 0.38 mA/cm^2^, 20 min, 7.60 C/cm^2^	Exp (1) Alpha (10 Hz) Exp (2) Theta (6 Hz)
Feurra^45^	Online	PPC: L-PPC	L- shoulder A	1	1 mA, 35 cm^2^, 0.03 mA/cm^2^, 15 min, 0.45 C/cm^2^	Theta (5 Hz), Alpha (10 Hz), Beta (20 Hz), Gamma (40 Hz)
Fusco^57^	Online	PFC: M-PFC	M-PCC	1	1.5 mA, 25 cm^2^, 0.06 mA/cm^2^, 4 min, 0.24 C/cm^2^	Delta (2 Hz), Theta (6 Hz), Alpha (11 Hz), Beta (21 Hz), Gamma (60 Hz)
Giustiniani^58^	Online Offline	PFC: L-M1	R-SO A	1	2 mA, 25 cm^2^, 0.08 mA/m^2^, 5 min, 0.40 C/cm^2^	Delta (1 Hz), Gamma (40 Hz)
Grabner^87^	Online	PFC: L-PFC	R-PFC	1	1 mA, 35 cm^2^, 0.03 mA/cm^2^, 30 min, 0.90 C/cm^2^	Alpha (10 Hz), Gamma (40 Hz)
Gutteling^88^	Online	PPC: Bi-POC	Vertex (Cz)	1	1 mA, 12 cm^2^, 0.08 mA/cm^2^, 25 min, 2.00 C/cm^2^	Alpha (10 Hz)
Hopfinger^89^	Online	PPC: R-PPC	Vertex (Cz)	1	2 mA, 25 cm^2^, 0.08 mA/cm^2^, 33 min, 2.64 C/cm^2^	Alpha (10 Hz), Gamma (40 Hz)
Hoy^59^	Offline	PFC: L-DLPFC	R-SO A	1	2 mA, 35 cm^2^, 0.06 mA/cm^2^, 20 min, 1.20 C/cm^2^	Gamma (40 Hz)
Janik^60^	Online	PFC: M-M1	R-POC	1	1 mA, 35 cm^2^, 0.03 mA/cm^2^, 16 min, 0.48 C/cm^2^	Gamma (40 Hz)
Jaušovec (BP)^61^	Offline	PPC: L-PPC	R-SO A	1	1.7 mA, 35 cm^2^, 0.05 mA/cm^2^, 15 min, 0.75 C/cm^2^	Theta (5 Hz)
Jaušovec (AP)^62^	Offline	PPC: L-PPC	R-SO A	1	1.5 mA, 35 cm^2^, 0.04 mA/cm^2^, 15 min, 0.60 C/cm^2^	Theta (5 Hz)
Javadi^90^	Online	PFC: L-DLPFC	L-Wrist	2	1.5 mA, 35 cm^2^, 0.04 mA/cm^2^, 16 min, 0.64 C/cm^2^	Gamma (60 Hz)
Kasten^91^	Online	PPC: M-POC	Vertex (Cz)	1	0.7 mA, 16 cm^2^, 0.04 mA/cm^2^, 20 min, 0.80 C/cm^2^	Alpha (10.5 ± 0.9 Hz)
Laczó^92^	Online	PPC: M-POC	Vertex (Cz)	1	1.5 mA, 16 cm^2^, 0.09 mA/cm^2^, 15 min, 1.35 C/cm^2^	Gamma (40 Hz)
Lang^63^	Offline	TC: R-FC	R-SO A + Bi-PPC + L-POC	1	2 mA, 4 cm^2^, 0.50 mA/cm^2^, 10 min, 5.00 C/cm^2^	Theta (6 Hz)
Loffler^64^	Online	PPC: M-POC	Vertex (Cz)	1	2 mA, 20 cm^2^, 0.10 mA/cm^2^, 30 min, 3.00 C/cm^2^	Gamma (40 Hz)
Luft^65^	Online Offline	TC: L-TC	Exp (1) Vertex (Cz), Exp (2) M-PFC	2	Exp (1) 1 mA, 25 cm^2^, 0.04 mA/cm^2^, 30 min, 1.20 C/cm^2^ Exp (3) 1 mA, 25 cm^2^, 0.04 mA/cm^2^, 25 min, 1.00 C/cm^2^	Exp (1) Alpha (10 Hz) Exp (2) Alpha (8–10 Hz)
Lustenberger^66^	Online	PFC: Bi-PFC	Vertex (Cz)	1	1 mA, 35 cm^2^, 0.03 mA/cm^2^, 30 min, 0.90 C/cm^2^	Exp (1) Alpha (10 Hz) Exp (2) Gamma (40 Hz)
Marchesotti^67^	Offline	TC: L-TC	L-TC + L-PPC + L-M1 + L-Mastoids A	3	1.1 mA, 4 cm^2^, 0.28 mA/cm^2^, 20 min, 5.60 C/cm^2^	Gamma (30 Hz)
Meier^68^	Online	Multi: L-PFC + L-PPC + R-TC	R-PFC + R-PPC +L-TC	1	1 mA, 1.2 cm^2^, 0.83 mA/cm^2^, 20 min, 16.0 C/cm^2^	Gamma (40 Hz)
Meiron^69^	Online	PFC: L-DLPFC	R-DLPFC	1	1 mA, 16 cm^2^, 0.06 mA/cm^2^, 20 min, 1.20 C/cm^2^	Theta (4.5 Hz)
Meng^70^	Offline	PPC: L-PPC	NR	1	2 mA, 3 cm^2^, 0.67 mA/cm^2^, 15 min, 10.5 C/cm^2^	Theta (6 Hz)
Moliadze^93^	Offline	PFC: Bi-PFC	NR	1	1 mA, 9 cm^2^, 0.11 mA/cm^2^, 20 min, 2.20 C/cm^2^	Alpha (10 Hz), Beta (16.8 Hz)
Neubauer^94^	Offline	PPC: L-PPC	Vertex (Cz)	1	1.5 mA, 35 cm^2^, 0.04 mA/cm^2^, 15 min, 0.60 C/cm^2^	Theta (5 Hz)
Nomura^71^	Offline	PFC: L-PFC	L-Wrist	2	1.5 mA, 35 cm^2^, 0.04 mA/cm^2^, 15 min, 0.60 C/cm^2^	Gamma (60 Hz)
Pahor^72^	Online	PPC: L-PPC	R-SO A	1	1.75 mA, 35 cm^2^, 0.05 mA/cm^2^, 15 min, 0.75 C/cm^2^	Theta (5 Hz)
Pahor^95^	Offline	PFC: Bi-DLPFC	NR	1	1.75 mA, 35 cm^2^, 0.05 mA/cm^2^, 15 min, 0.75 C/cm^2^	Alpha (10.95 ± 0.98 Hz)
Polanía^73^	Online	Multi: L-PFC + L-PPC	Vertex (Cz)	1	1 mA, 25 cm^2^, 0.04 mA/cm^2^, 14 min, 0.56 C/cm^2^	Theta (6 Hz), Gamma (35 Hz)
Polanía^74^	Online	Multi: M-PFC + PPC	R-Shoulder	1	2 mA, 35 cm^2^, 0.06 mA/cm^2^, 18 min, 1.08 C/cm^2^	Gamma (55 Hz)
Pollok^75^	Online	PFC: L-M1	R-SO A	1	1 mA, 35 cm^2^, 0.03 mA/cm^2^, 12 min, 0.36 C/cm^2^	Alpha (10 Hz), Beta (20 Hz)
Reinhart^96^	Online Offline	Exp (1, 3) PFC: M-PFC + R-PFC Exp (2) PFC: M-PFC + L-PFC	Surrounding	1	1 mA, 4 cm^2^, 0.25 mA/cm^2^, 20 min, 5.00 C/cm^2^	Exp (1) Theta (6 Hz), Gamma (35 Hz) Exp (2, 3) Theta (6 Hz)
Riecke^97^	Online	TC: Bi-TC	Vertex (Cz)	1	0.8 mA, 25 cm^2^, 0.03 mA/cm^2^, 40 min, 1.20 C/cm^2^	Theta (4 Hz)
Riecke^98^	Online	TC: Bi-TC	Vertex (Cz)	1	0.9 mA, 25 cm^2^, 0.04 mA/cm^2^, 36 min, 1.44 C/cm^2^	Theta (4 Hz)
Santarnecchi^76^	Online	PFC: L-DLPFC	Vertex (Cz)	1	0.75 mA, 35 cm^2^, 0.02 mA/cm^2^, 48 min, 0.96 C/cm^2^	Theta (5 Hz), Alpha (10 Hz), Beta (20 Hz), Gamma (40 Hz)
Santarnecchi^77^	Online	PFC: L-DLPFC	Vertex (Cz)	1	1.5 m, 25 cm^2^, 0.06 mA/cm^2^, 30 min, 1.80 C/cm^2^	Theta (5 Hz), Gamma (40 Hz)
Santarnecchi^78^	Online	PPC: R-PPC TC: R-TC	R-PFC + L-PPC + L-TC L-PFC + L-PPC + L-TC	1	2 mA, 35 cm^2^, 0.06 mA/cm^2^, 9 min, 0.50 C/cm^2^	Alpha (10 Hz) Gamma (40 Hz)
Schuhmann^79^	Online	PPC: L-PPC	L-PPC	1	1 mA, 2.1 cm^2^, 0.48 mA/cm^2^, 35 min. 16.8 C/cm^2^	Alpha (10 Hz)
Sela^80^	Online	PFC: L-DLPFC	L-TC	1	1 mA, 25 cm^2^, 0.04 mA/cm^2^, 15 min, 0.60 C/cm^2^	Theta (6.5 Hz)
Strüber^99^	Online	Exp (1, 2) PPC: L-POC Exp (3) PPC: B-POC	Exp (1, 2) R-POC Exp (3) B-PFC	1	Exp (1, 2) 0.76 mA, 35 cm^2^, 0.02 mA/cm^2^, 15 min, 0.30 C/cm^2^ Exp (3) 1.3 mA, 15.21 cm^2,^ 0.09 mA/cm^2^, 15 min, 1.35 C/cm^2^	Theta (6 Hz), Gamma (40 Hz)
Tseng ^100^	Online	Multi: L-TC + L-PPC	R-Cheek	1	1.5 mA, 25 cm^2^, 0.06 mA/cm^2^, 20 min, 1.20 C/cm^2^	Gamma (40 Hz)
Tseng^101^	Online	PPC: Bi-PPC	L-Cheek	1	1.6 mA, 16 cm^2^, 0.10 mA/cm^2^, 20–24 min, 2.20 C/cm^2^	Theta (6 Hz)
Violante^81^	Online	Multi: R-PFC + R-PPC	R-TC	2	1 mA, 5 cm^2^, 0.20 mA/cm^2^, 26.5 min, 5.30 C/cm^2^	Theta (6 Hz)
Vosskuhl^46^	Online Offline	PFC: M-PFC	M-PPC	3	0.8 mA, 35 cm^2^, 0.02 mA/cm^2^, 18 min, 0.36 C/cm^2^	Theta (3.7–4.6 Hz)
Wischnewski^82^	Online	PFC: R-PFC + L-PFC	NR	1	1 mA, 35 cm^2^, 0.03 mA/cm^2^, 11 min, 0.33 C/cm^2^	Theta (6 Hz)
Wöstmann^102^	Online	Multi: L-TC + L-PPC	NR	1	1 mA, 3 cm^2^, 0.34 mA/cm^2^, 25 min, 8.50 C/cm^2^	Alpha (10 Hz), Gamma (47.1 Hz)
Wynn^83^	Online	PPC: Bi-PPC	Vertex (Cz)	1	2 mA, 25 cm^2^, 0.08 mA/cm^2^, 30 min, 2.40 C/cm^2^	Theta (3.5 Hz), Alpha (8 Hz)
Zavecz^103^	Online	Multi: M-PFC + M-PPC	NR	4	1 mA, 25 cm^2^, 0.04 mA/cm^2^, 20 min, 0.80 C/cm^2^	Theta (6 Hz)
Zoefel^104^	Online	TC: L-TC	L-PFC	1	1.7 mA, 9 cm^2^, 0.19 mA/cm^2^, 30 min, 5.70 C/cm^2^	Delta (3.125 Hz)
Zoefel^105^	Online	Exp (1) TC: L-TC, Exp (2) TC: Bi-TC	Exp (1) L-PFC, Exp (2) Surrounding	2	Exp (1) 1.2 mA, 9 cm^2^, 0.13 mA/cm^2^, 30 min, 3.90 C/cm^2^ Exp (2) 1.7 mA, 9 cm^2^, 0.19 mA/cm^2^, 30 min, 5.70 C/cm^2^	Delta (3.125 Hz)

*A* area, *AP* published in the Acta Psychologica, *Bi* bilateral hemisphere, *BP* published in the Biological Psychology, *C* cortex, *DLPFC* dorsolateral–prefrontal cortex, *FC* fusiform cortex, *Hz* hertz, *IFG* inferior frontal gyrus, *L* left, *M* medial, *Multi* multiple regions, *NR* not reported, *M1* primary motor cortex, *OFC* orbitofrontal cortex, *PARC* parietal cortex, *PFC* prefrontal cortex, *POC* posterior occipital cortex, *PPC* posterior parietal cortex, *R* right, *SO* supraorbital, *TC* temporal cortex.

Regarding the potential side effects of tACS protocols, 11 studies confirmed that participants did not experience any side effects. Twenty-three studies reported that some participants experienced side effects: (a) discomfort = three studies, (b) itching = 10 studies, (c) mild headache = four studies, (d) tingling = 11 studies, (e) tiredness = three studies, (f) phosphene (flickering) = eight studies, (g) attention difficulties = six studies, (h) dizziness = one study, (i) pain (e.g., pinch, burning, heat, shock-like sensations, pricking) = six studies, and (j) other side effects = three studies (e.g., fatigue, tiring, and anxiety)^54–57,63,64,66,67,70,73,75–77,80,82,83,85,87,88,92,93,102,104^. In the 34 studies, ~46.2% of participants (i.e., number of participants from studies that reported the presence of side effects / total number of participants from studies that reported presence or absence of side effects × 100) may experience potential side effects of tACS protocols (Supplementary Table 1). However, the remaining 22 studies failed to mention whether participants experienced side effects.

### Cognitive function assessments

Thirty-eight out of 56 qualified studies reported cognitive performance variables and six studies showed cognition-related reaction time variables. The remaining twelve studies reported both cognitive performance and reaction time variables. Taken together, 50 out of 56 qualified studies reported cognitive performance variable comparisons and 18 out of 56 qualified studies reported cognition-related reaction time variable comparisons (Table 3).

**Table 3:** Specific cognitive function assessment and cognitive domains.

Study	Cognitive assessments	Cognitive task	Cognitive domains
Alekseichuk^52^	Performance (memory performance %) Reaction time (reaction time)	Two-back visual-spatial task	Executive function/complex attention
Alekseichuk^84^	Performance (correct %)	Memory recognition task	Learning and memory
Ambrus^53^	Performance (number of correctly recalled words)	Word-pair learning task	Learning and memory
Antonenko^54^	Performance (correct %)	Language learning paradigm	Learning and memory
Brauer^55^	Performance (number of error) Reaction time (reaction time)	Go/Nogo task	Executive function/complex attention
Braun^85^	Performance (hits %)	Memory performance for words	Executive function/complex attention
Brignani^56^	Performance (accuracy)	Visual detection and discrimination task	Perceptual-motor function
Deng^86^	Performance (correct %)	Selective auditory attention task	Executive function/complex attention
Feurra^45^	Performance (digit span forward scores)	Digit forward	Executive function/complex attention
Fusco^57^	Performance (behavioral adaptation)	Flanker task	Executive function/complex attention
Giustiniani^58^	Reaction time (mean RT)	Serial reaction time task	Learning and memory
Grabner^87^	Performance (scores)	Verbal creativity task	Executive function/complex attention
Gutteling^88^	Performance (updating gain)	Whole-body motion updating task	Perceptual-motor function
Hopfinger^89^	Reaction time (mean RT)	Visual attention task	Executive function/complex attention
Hoy^59^	Performance (d-prime) Reaction time (reaction time)	N-back task	Executive function/complex attention
Janik^60^	Performance (correct responses %)	Cambridge face perception identity task	Perceptual-motor function
Jaušovec (BP)^61^	Performance (memory capacity scores)	Visual-array comparison task	Executive function/complex attention
Jaušovec (AP)^62^	Performance (memory capacity scores)	Forward and backward corsi block-tapping task	Executive function/complex attention
Javadi^90^	Performance (correct %)	Declarative memory task	Executive function/complex attention
Kasten^91^	Performance (performance change %) Reaction time (reaction time change %)	Mental rotation task	Perceptual-motor function
Laczó^92^	Performance (false-choice trial)	Four alternative forced choice task	Perceptual-motor function
Lang^63^	Performance (correct associative memory)	Face and scene task	Perceptual-motor function
Loffler^64^	Performance (mean error) Reaction time (reaction time)	Visual two-choice task	Perceptual-motor function
Luft^65^	Performance (creativity)	Remote associate task/Divergent thinking task	Executive function/complex attention
Lustenberger^66^	Performance (creativity index)	Creative thinking task	Executive function/complex attention
Marchesotti^67^	Performance (performance)	Phonemic awareness task	Executive function/complex attention
Meier^68^	Performance (laterality index)	Dichotic listening task	Perceptual-motor function
Meiron^69^	Performance (memory accuracy) Reaction time (reaction time)	N-back task	Executive function/complex attention
Meng^70^	Performance (recognition)	Face and scene task	Perceptual-motor function
Moliadze^93^	Performance (number of error) Reaction time (mean RT)	Phonological decision task	Executive function/complex attention
Neubauer^94^	Performance (performance)	Raven’s progressive matrices test	Executive function/complex attention
Nomura^71^	Performance (hits ratio)	Episodic memory task	Learning and memory
Pahor^72^	Performance (fluid intelligence scores)	Fluid intelligence task	Executive function/complex attention
Pahor^95^	Performance (score)	Raven’s progressive matrices task	Executive function/complex attention
Polanía^73^	Reaction time (reaction time)	Delayed letter discrimination task	Executive function/complex attention
Polanía^74^	Performance (corrects and accuracy) Reaction time (reaction time)	Decision-making task	Executive function/complex attention
Pollok^75^	Reaction time (learning index reaction time)	Serial reaction time task	Learning and memory
Reinhart^96^	Performance (mean error)	Time-estimation task	Executive function/complex attention
Riecke^97^	Performance (false alarm rate)	Naturalistic listening task	Perceptual-motor function
Riecke^98^	Performance (performance)	Phoneme-categorization task	Language
Santarnecchi^76^	Performance (accuracy %) Reaction time (reaction time)	Fluid intelligence task	Executive function/complex attention
Santarnecchi^77^	Performance (accuracy %) Reaction time (reaction time)	Abstract-reasoning task/Change- localization working memory task	Executive function/complex attention
Santarnecchi^78^	Performance (accuracy %) Reaction time (reaction time)	Insight task	Executive function/complex attention
Schuhmann^79^	Reaction time (reaction time)	Endogenous attention task	Perceptual-motor function
Sela^80^	Performance (number of adjusted pumps)	Balloon analog risk task	Executive function/complex attention
Strüber^99^	Performance (motion dominance index)	Stroboscopic alternative motion task	Perceptual-motor function
Tseng^100^	Performance (d-index)	Change detection task	Executive function/complex attention
Tseng^101^	Performance (Pashler’s K)	Change detection task	Executive function/complex attention
Violante^81^	Reaction time (reaction time)	Choice reaction time task	Executive function/complex attention
Vosskuhl^46^	Performance (correctly answered scores)	Digit span task/N-back task	Executive function/complex attention
Wischnewski^82^	Performance (average probability high risk)	Reinforcement learning task	Learning and memory
Wöstmann^102^	Performance (hits %)	Dichotic listening task	Executive function/complex attention
Wynn^83^	Performance (d-prime)	Recognition memory task	Learning and memory
Zavecz^103^	Performance (score) Reaction time (reaction time)	Alternating serial reaction time task	Learning and memory
Zoefel^104^	Performance (d-prime)	Detection task	Executive function/complex attention
Zoefel^105^	Performance (correct %)	Word report task	Executive function/complex attention

*AP* published in the Acta Psychologica, *BP* published in the Biological Psychology, *RT* reaction time.

For cognitive performance variables, specific measurements were: (a) accuracy = six studies, (b) correctness (e.g., correctly recalled words, correct response, and correct associative memory) = eight studies, (c) creativity index: two studies, (d) d-prime = three studies, (e) number of errors = four studies, (f) scores (e.g., digit span forward scores, memory capability scores, fluid intelligence scores, and correctly answered scores) = eight studies, and (g) others (e.g., average probability, behavioral adaptation, d-index, false-choice trial, hit ratio, laterality index, memory performance, motion dominance index, number of adjusted pumps, Pashler’s K, performance change rate, recognition, and updating gain) = 19 studies.

In this study, specific cognitive domains included: (a) perceptual-motor function (e.g., visual detection, Cambridge face perception task, and face and scene task) = 12 studies, (b) learning and memory (e.g., memory recognition task, word-pair learning task, and language learning task) = nine studies, (c) executive function / complex attention (e.g., n-back task, digit span task, and change detection task): = 34 studies, and (d) language (e.g., phoneme-categorization task): = one study.

### Specific comparisons for meta-analysis

For meta-analysis procedures, we acquired specific comparisons from each included study because of different experiments, timing (i.e., online and offline), and frequency bands (i.e., delta, theta, alpha, beta, gamma, and ripple) of tACS protocols. Twenty-eight out of 50 studies that used cognitive performance variables reported one comparison, and 22 studies reported multiple comparisons (i.e., 13 studies reported two comparisons, two studies reported three comparisons, five studies reported four comparisons, one study reported five comparisons, and one study showed eight comparisons). For 18 studies that used cognition-related reaction time variables, nine studies reported one comparison and nine studies reported multiple comparisons (i.e., six studies reported two comparisons, one study reported three comparisons, and two studies reported four comparisons). Taken together, the meta-analysis focused on 93 total cognitive performance variable comparisons from the 50 studies and 32 total cognition-related reaction time variable comparisons from the 18 studies.

### Methodological quality assessments

**Fig. 2:**
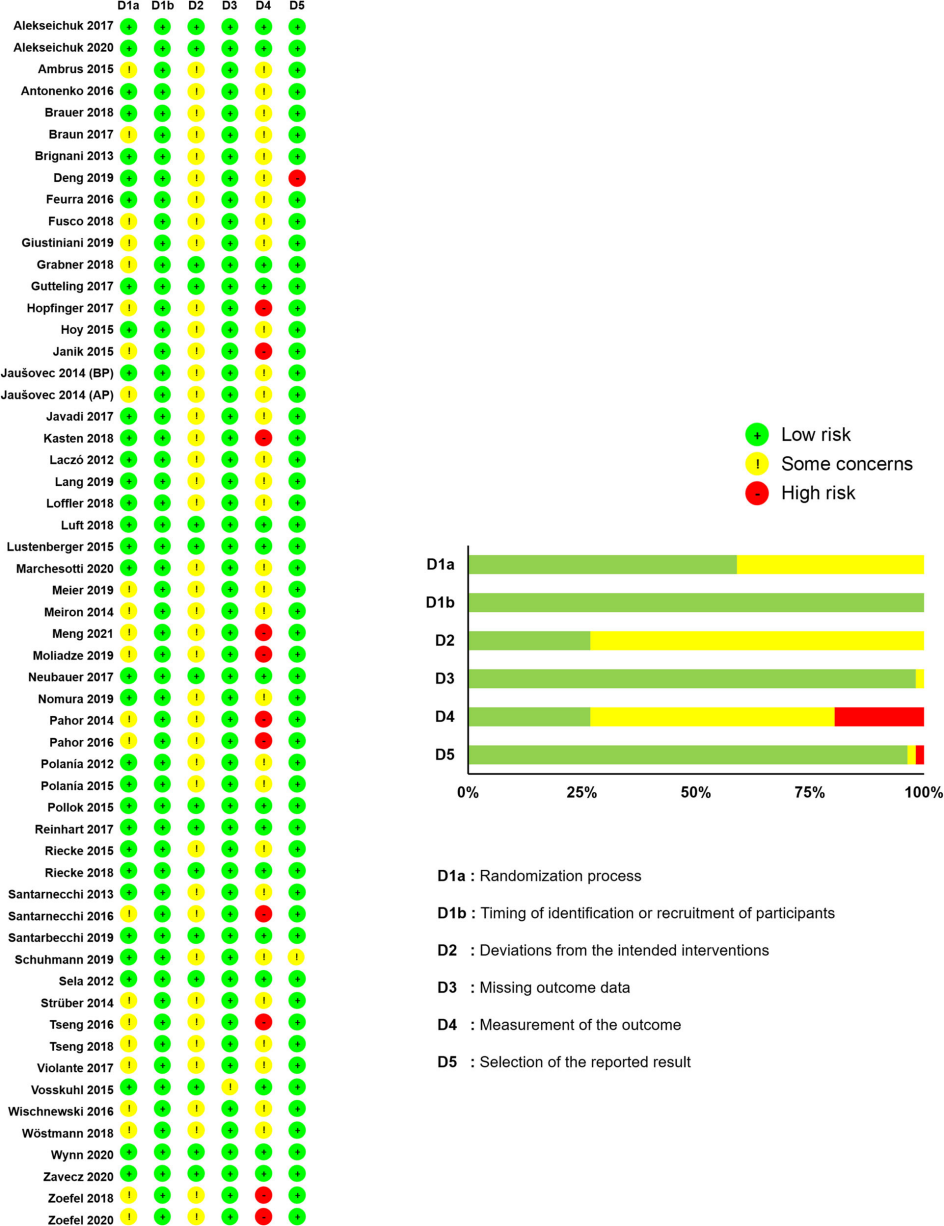
Methodological quality assessment. The Cochrane risk of bias assessment reveals potential methodological concerns.

The Cochrane risk of bias assessment showed three potential methodological concerns including (a) randomized process, (b) deviations from intended interventions, and (c) measurements of the outcome. Especially, 23 included studies failed to either mention a specific randomization process or randomly assign the tACS conditions, and 41 out of 56 studies did not mention the blinding of experimenters or assessors. However, we confirmed that the current meta-analysis showed a low level of risk bias in (a) timing of identification or recruitment of participants, (b) missing outcome data, and (c) selection of the reported result domains (Fig. 2).

### Meta-analytic findings on cognitive performance

The random-effects meta-analysis on 93 total comparisons from the 50 studies identified a significant low overall effect of tACS protocols on cognitive performance improvements (*SMD* = 0.161; *SE* = 0.027; 95% CI = 0.109–0.214; Z = 6.038; *P* < 0.001). The heterogeneity tests revealed lower level of variability across the 93 comparisons (*Q*-statistics = 130.256 and *P* = 0.005; *I*^2^ = 29.4%), and the publication bias was the relatively asymmetrical distribution of individual effect sizes: (1) a revised funnel plot with 7 imputed values (Supplementary Fig. 1) and (2) Egger’s regression intercept (*β*_0_) = 1.57 and *P* = 0.001.

**Fig. 3:**
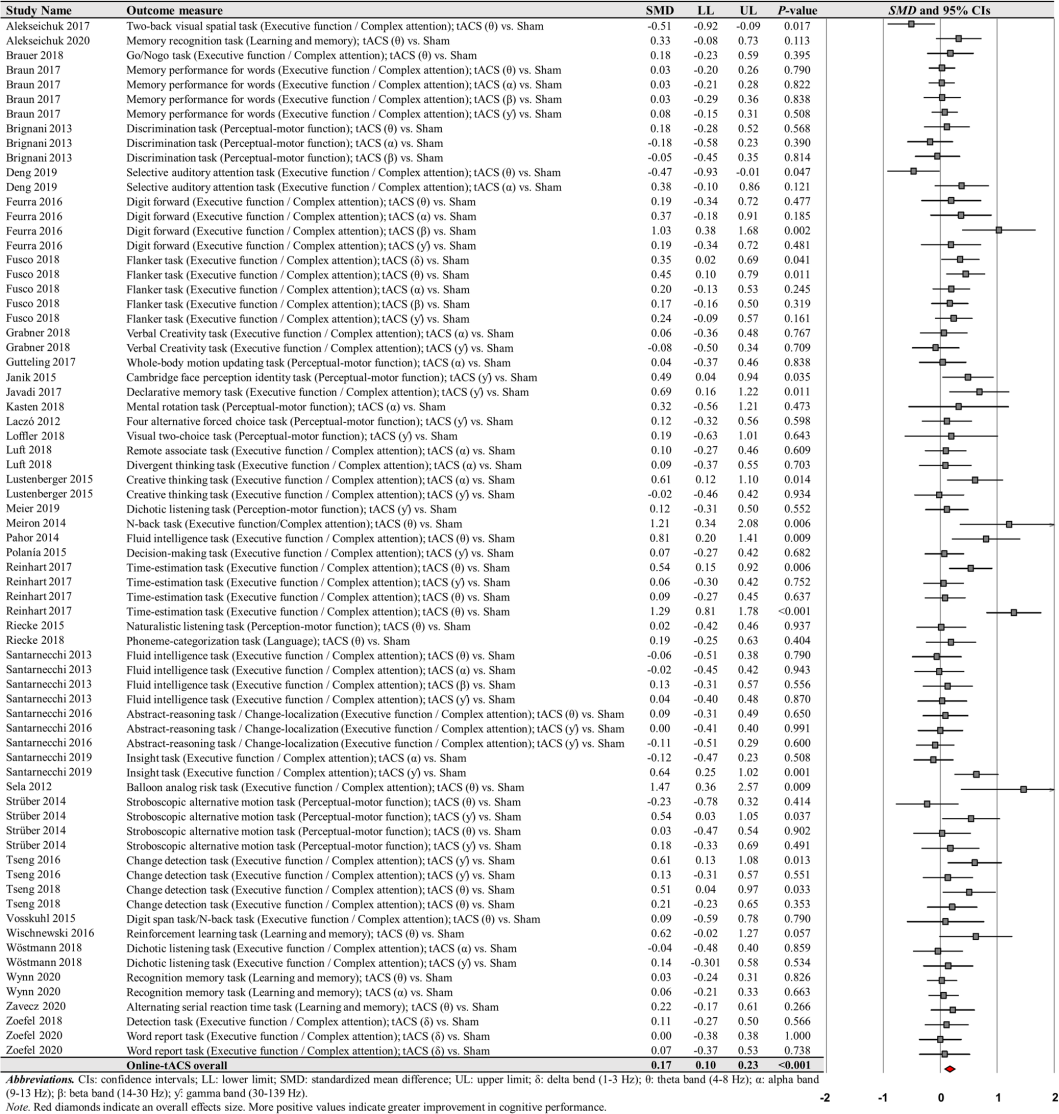
Cognitive performance comparisons after online-tACS. Meta-analytic findings show potential effects of online-tACS protocols on changes in cognitive performances.

**Fig. 4:**
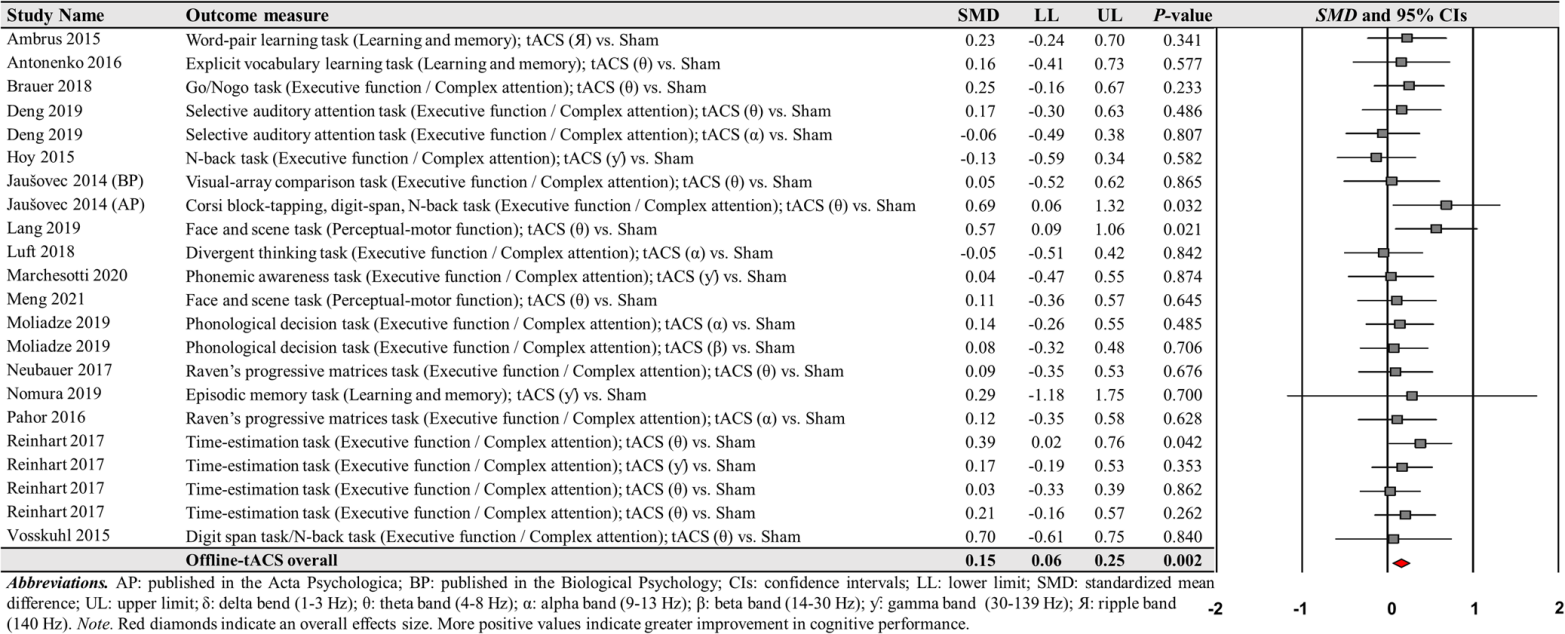
Cognitive performance comparisons after offline-tACS. Meta-analytic findings show potential effects of offline-tACS protocols on changes in cognitive performances.

The first moderator variable analysis for comparing the effects of online-tACS versus offline-tACS on changes in cognitive performance showed significant treatment effects: (a) 71 online-tACS comparisons from the 38 studies: *SMD* = 0.168; *SE* = 0.033; 95% CI = 0.104–0.233; Z = 5.138; *P* < 0.001; *Q*-statistics = 118.535 with *P* < 0.001; *I*^2^ = 41.0% (Fig. 3) and (b) 22 offline-tACS comparisons from the 17 studies: *SMD* = 0.153; *SE* = 0.049; 95% CI = 0.056–0.250; Z = 3.092; *P* = 0.002; *Q*-statistics = 11.715 with *P* = 0.947; *I*^2^ = 0.0% (Fig. 4). These findings indicate that tACS protocols showed significant improvements in cognitive performance regardless of stimulation timing.

**Fig. 5:**
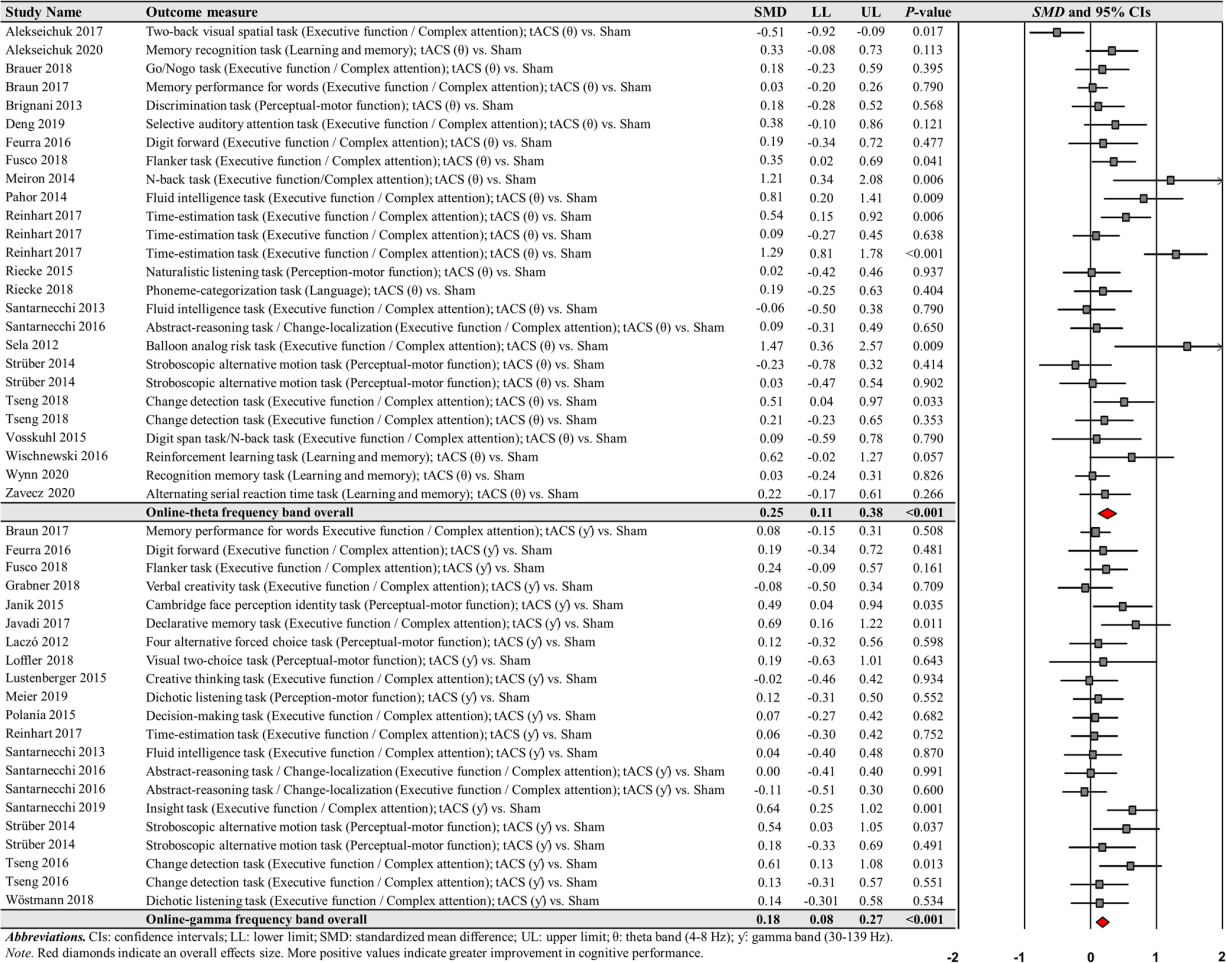
Cognitive performance comparisons after online-tACS with theta and gamma frequency bands. Meta-analytic findings show potential effects of online-tACS protocols with theta and gamma frequency bands on changes in cognitive performances.

For online-tACS comparisons, the second moderator variable analysis for comparing the effects of different frequency bands (i.e., delta vs. theta vs. alpha vs. beta vs. gamma) of tACS protocols showed significant positive effects of theta and gamma frequency bands on cognitive performance: (a) 26 theta frequency band comparisons from the 22 studies: *SMD* = 0.247; *SE* = 0.069; 95% CI = 0.111–0.383; Z = 3.556; *P* < 0.001; *Q*-statistics = 62.079 with *P* < 0.001; *I*^2^ = 59.7% and (b) 21 gamma frequency band comparisons from the 18 studies: *SMD* = 0.175; *SE* = 0.049; 95% CI = 0.078–0.272; Z = 3.547; *P* < 0.001; *Q*-statistics = 23.442 with *P* = 0.268; *I*^2^ = 14.7% (Fig. 5).

**Fig. 6:**
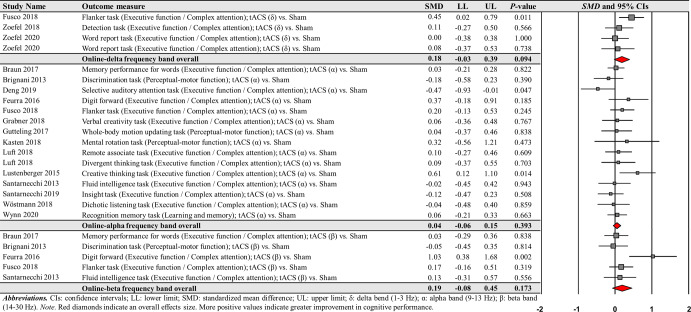
Cognitive performance comparisons after online-tACS with delta, alpha, and beta frequency bands. Meta-analytic findings show no significant effects of online-tACS protocols with delta, alpha, and beta frequency bands on changes in cognitive performances.

However, the analyses revealed no significant effects of delta, alpha, and beta frequency bands on cognitive performance improvements: (a) four delta frequency band comparisons from the three studies: *SMD* = 0.178; *SE* = 0.106; 95% CI = −0.030–0.387; Z = 1.675; *P* = 0.094; *Q*-statistics = 3.525 with *P* = 0.318; *I*^2^ = 14.9%, (b) 15 alpha frequency band comparisons from the 14 studies: *SMD* = 0.044; *SE* = 0.052; 95% CI = −0.058–0.146; Z = 0.854; *P* = 0.393; *Q*-statistics = 14.800 with *P* = 0.392; *I*^2^ = 5.4%, and (c) five beta frequency band comparisons from the five studies: *SMD* = 0.185; *SE* = 0.136; 95% CI = −0.081–0.450; Z = 1.363; *P* = 0.173; *Q*-statistics = 8.516 with *P* = 0.074; *I*^2^ = 53.0% (Fig. 6).

**Fig. 7:**
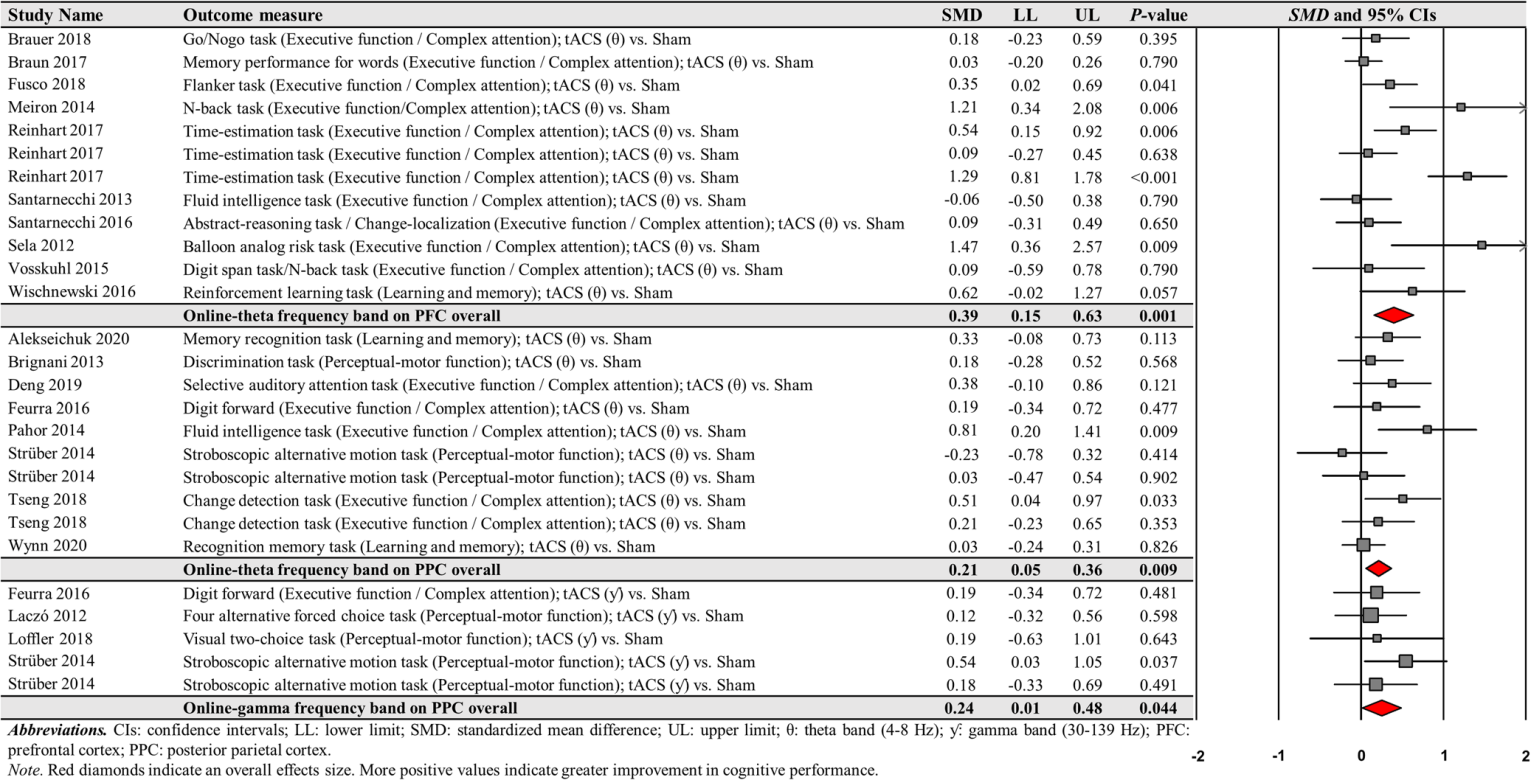
Cognitive performance comparisons after online-tACS with theta and gamma frequency bands across targeted brain regions. Meta-analytic findings show potential effects of online-tACS protocols with theta frequency band on PFC and PPC and online-tACS protocols with gamma frequency band on PFC.

For the comparisons of each frequency band of online-tACS protocols, the third moderator variable analysis examined specific changes in cognitive performances among different targeted brain regions, respectively. The analysis revealed significant positive effects for the following conditions: (a) 12 PFC in the theta frequency band comparisons from 10 studies: *SMD* = 0.389; *SE* = 0.122; 95% CI = 0.149–0.629; Z = 3.180; *P* = 0.001; *Q*-statistics = 37.850 with *P* < 0.001; *I*^2^ = 70.9%, (b) 10 PPC in the theta frequency band comparisons from eight studies: *SMD* = 0.206; *SE* = 0.078; 95% CI = 0.052–0.359; Z = 2.627; *P* = 0.009; *Q*-statistics = 10.930 with *P* = 0.281; *I*^2^ = 17.658%, and (c) five PPC in the gamma frequency band comparisons from four studies: *SMD* = 0.243; *SE* = 0.120; 95% CI = 0.007–0.479; Z = 2.018; *P* = 0.044; *Q*-statistics = 1.747 with *P* = 0.782; *I*^2^ = 0.0% (Fig. 7). We found no significant changes in cognitive performance variables for the remaining conditions (Supplementary Table 2).

**Fig. 8:**
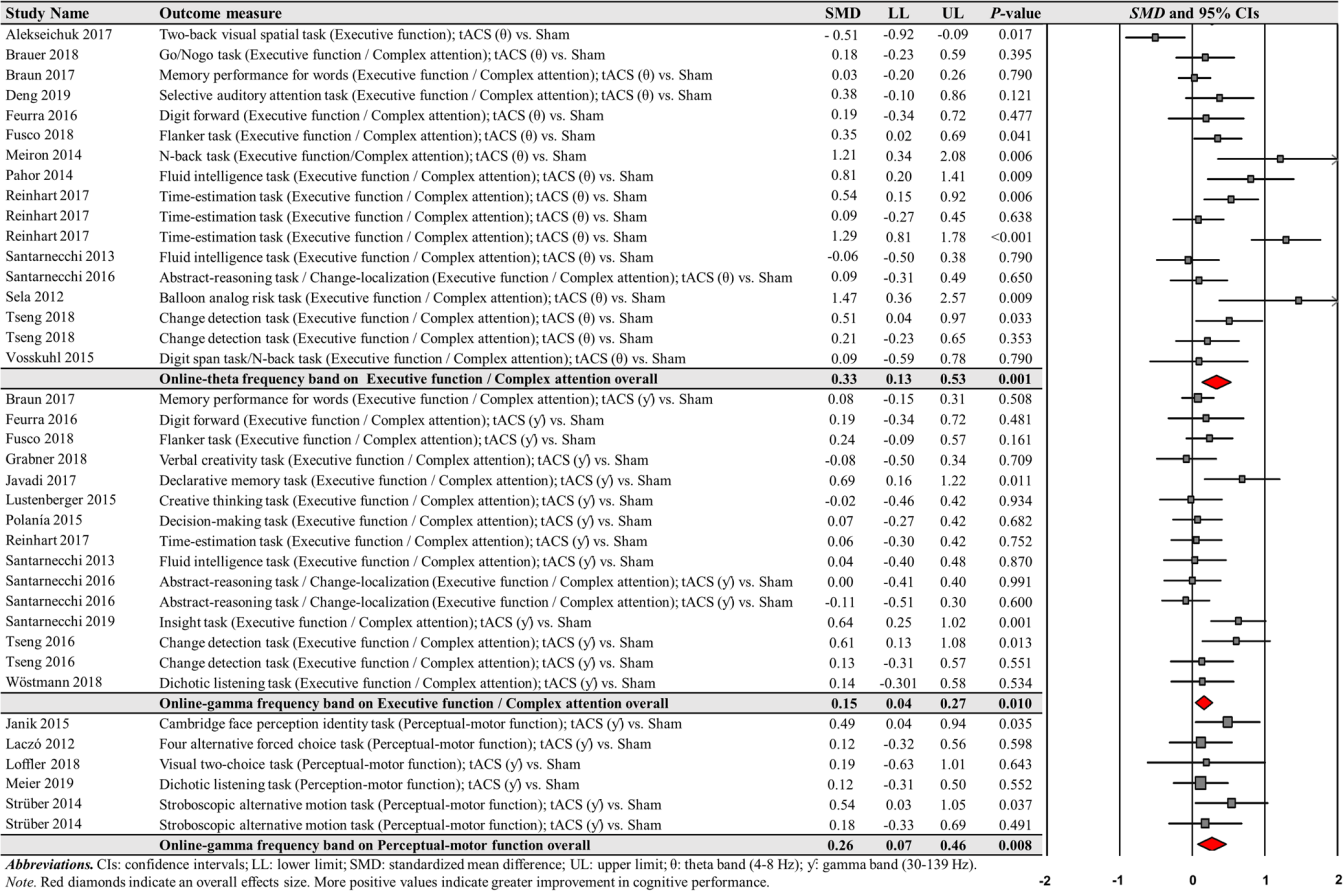
Cognitive performance comparisons after online-tACS with theta and gamma frequency bands across cognitive domains. Meta-analytic findings show potential effects of online-tACS protocols with theta frequency band on changes in executive function / complex attention and online-tACS protocols with gamma frequency band on changes in executive function / complex attention and perceptual-motor function.

For the comparisons of each frequency band of online-tACS protocols, the fourth moderator variable analysis investigated different changes in cognitive performances based on cognitive domains, respectively. The analysis revealed significant positive effects for the following conditions: (a) 17 executive function / complex attention in the theta frequency band comparisons from 14 studies: *SMD* = 0.325; *SE* = 0.102; 95% CI = 0.125–0.526; Z = 3.180; *P* = 0.001; *Q*-statistics = 54.206 with *P* = 0.001; *I*^2^ = 70.5%, (b) 15 executive function / complex attention in the gamma frequency band comparisons from 13 studies: *SMD* = 0.154; *SE* = 0.060; 95% CI = 0.036– 0.271; Z = 2.564; *P* = 0.010; *Q*-statistics = 19.114 with *P* = 0.161; *I*^2^ = 26.8%, and (c) six perceptual-motor function in the gamma frequency band comparisons from five studies: *SMD* = 0.264; *SE* = 0.100; 95% CI = 0.068–0.460; Z = 2.635; *P* = 0.008; *Q*-statistics = 3.198 with *P* = 0.669; *I*^2^ = 0.0% (Fig. 8). We found no significant changes in cognitive performance variables for the remaining conditions (Supplementary Table 3).

**Fig. 9:**
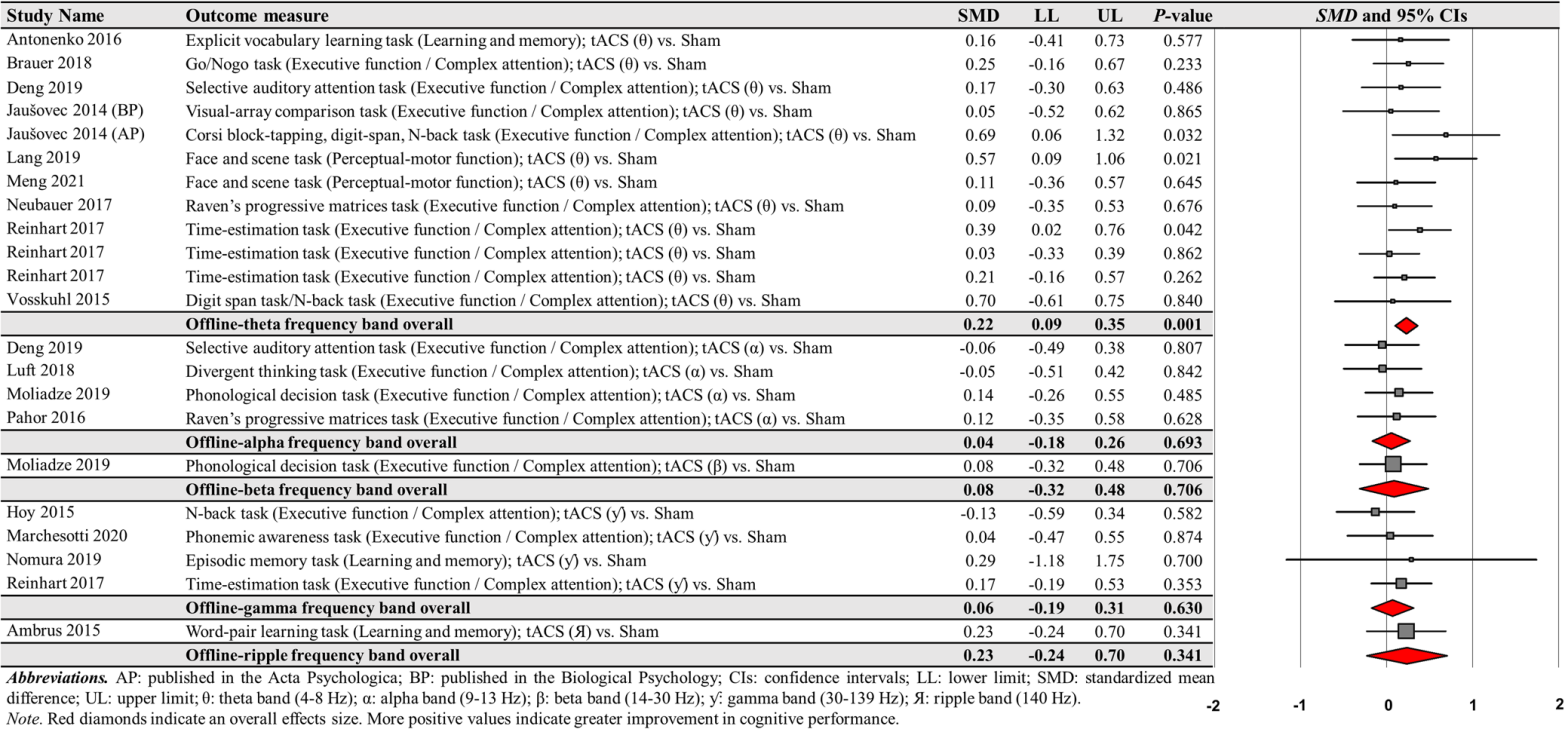
Cognitive performance comparisons after offline-tACS with specific frequency bands. Meta-analytic findings show potential effects of offline-tACS protocols with theta frequency band on changes in cognitive performances.

For offline-tACS comparisons, the moderator variable analysis on 12 theta frequency band comparisons from the 10 studies showed significant positive effects on cognitive performance: *SMD* = 0.221; *SE* = 0.067; 95% CI = 0.090–0.352; Z = 3.298; *P* = 0.001; *Q*-statistics = 7.183 with *P* = 0.784; *I*^2^ = 0.0% (Fig. 9). However, the analyses showed no significant effects on alpha, beta, gamma, and ripple frequency bands on cognitive performance improvements: (a) four alpha frequency band comparisons form the four studies: *SMD* = 0.044; *SE* = 0.112; 95% CI = −0.175–0.264; Z = 0.394; *P* = 0.693; *Q*-statistics = 0.667 with *P* = 0.881; *I*^2^ = 0.0% and (b) four gamma frequency band comparisons from the four studies: *SMD* = 0.060; *SE* = 0.125; 95% CI = −0.185–0.305; Z = 0.482; *P* = 0.630; *Q*-statistics = 1.107 with *P* = 0.775; *I*^2^ = 0.0%.

**Fig. 10:**
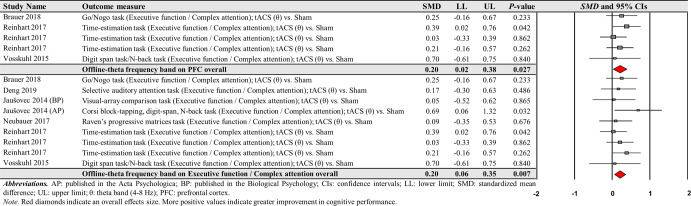
Cognitive performance comparisons after offline-tACS with theta frequency band across targeted brain regions and cognitive domains. Meta-analytic findings show potential effects of offline-tACS protocols with theta frequency band on PFC and executive function / complex attention.

For the comparisons of each frequency band of offline-tACS protocols, the third moderator variable analysis that examined specific changes in cognitive performances among different targeted brain regions revealed significant positive effect for the following condition: five PFC in the theta frequency band comparisons from three studies: *SMD* = 0.204; *SE* = 0.092; 95% CI = 0.023– 0.384; Z = 2.211; *P* = 0.027; *Q*-statistics = 2.009 with *P* = 0.734; *I*^2^ = 0.0% (Fig. 10). The fourth moderator variable analysis that investigated potential different treatment effects based on cognitive domains showed significant positive effect for the following condition: nine executive function / complex attention in the theta frequency band comparisons from seven studies: *SMD* = 0.204; *SE* = 0.075; 95% CI = 0.056–0.351; Z = 2.711; *P* = 0.007; *Q*-statistics = 4.855 with *P* = 0.773; *I*^2^ = 0.0% (Fig. 10). We found no significant changes in cognitive performance variables for the remaining conditions (Supplementary Tables 4 and 5).

### Meta-analytic findings on cognition-related reaction time

The random-effects model meta-analysis on 32 total comparisons from the 18 studies failed to demonstrate a significant effect of tACS protocols on the reaction time (*SMD* = 0.062; *SE* = 0.060; 95% CI = −0.055–0.180; Z = 1.038; *P* = 0.229) with moderate heterogeneity levels of variability across the 32 comparisons (*Q*-statistics = 66.181 and *P* < 0.001; *I*^2^ = 53.2%), and publication bias was the relatively symmetrical distribution of individual effect size: (1) a revised funnel plot with 8 imputed values and (2) Egger’s regression intercept (*β*_0_) = 0.92 and *P* = 0.465 (Supplementary Fig. 2).

**Fig. 11:**
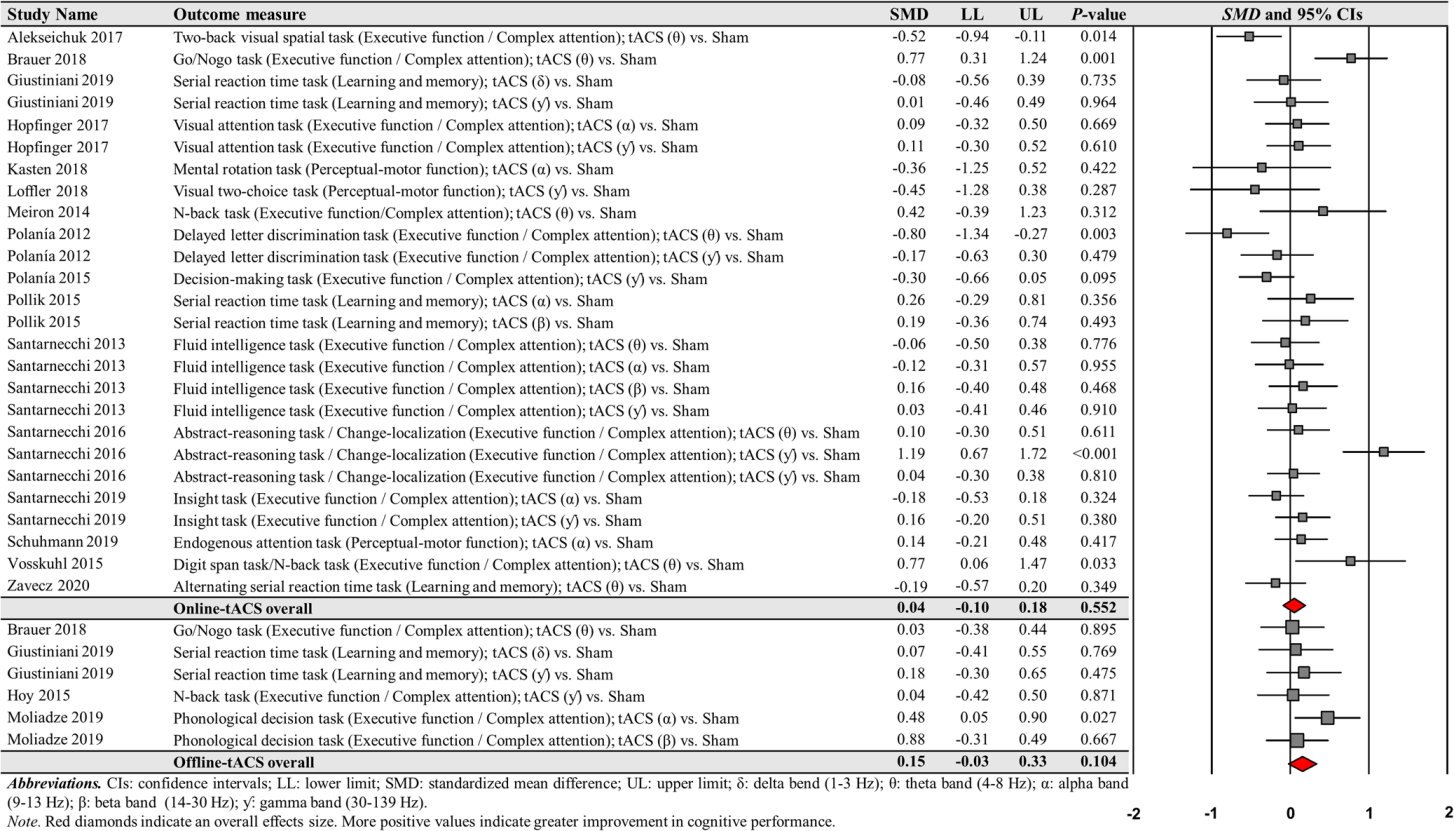
Cognition-related reaction time comparisons after online- and offline-tACS. Meta-analytic findings show no significant effects of online- and offline-tACS protocols on changes in cognition-related reaction time.

The moderator variable analysis for comparing effects of online-tACS versus offline-tACS on changes in cognition-related reaction time revealed no significant treatment effects: (a) 26 online-tACS comparisons from the 15 studies: *SMD* = 0.043; *SE* = 0.072; 95% CI = −0.098–0.184; Z = 0.595; *P* = 0.552; *Q*-statistics = 61.730 with *P* < 0.001; *I*^2^ = 59.5% and (b) six offline-tACS comparisons from the four studies: *SMD* = 0.148; *SE* = 0.091; 95% CI = −0.031–0.327; Z = 1.625; *P* = 0.104; *Q*-statistics = 3.058 with *P* = 0.691; *I*^2^ = 0.0% (Fig. 11).

For online-tACS comparisons, moderator variable analysis comparing the effects of different frequency bands (i.e., delta vs. theta vs. alpha vs. beta vs. gamma) of tACS protocols failed to show any significant positive effects on cognition-related reaction time (Supplementary Fig. 3): (a) eight theta frequency band comparisons from the eight studies: *SMD* = 0.029; *SE* = 0.189; 95% CI = −0.342–0.399; Z = −0.151; *P* = 0.880; *Q*-statistics = 32.153 with *P* < 0.001; *I*^2^ = 78.2%, (b) six alpha frequency band comparisons from the six studies: *SMD* = 0.020; *SE* = 0.090; 95% CI = −0.155–0.196; Z = −0.228; *P* = 0.820; *Q*-statistics = 3.266 with *P* = 0.659; *I*^2^ = 0.0%, (c) two beta frequency band comparisons from the two studies: *SMD* = 0.175; *SE* = 0.175; 95% CI = −0.169–0.518; Z = 0.995; *P* = 0.320; *Q*-statistics = 0.006 with *P* = 0.937; *I*^2^ = 0.0%, and (d) nine gamma frequency band comparison from the eight studies: *SMD* = 0.077; *SE* = 0.131; 95% CI = −0.180–0.333; Z = 0.587; *P* = 0.557; *Q*-statistics = 24.787 with *P* = 0.002; *I*^2^ = 67.7%. For the comparisons of each frequency band of online-tACS protocols, the third (different targeted brain regions) and fourth (different cognitive domains) moderator variable analyses identified no significant effects on cognition-related reaction time (Supplementary Tables 6 and 7).

For offline-tACS comparisons, moderator variable analysis comparing the effects of the different frequency band (i.e., delta vs. theta vs. alpha vs. beta vs. gamma) of tACS protocols failed to show any significant positive effects on cognition-related reaction time (Supplementary Fig. 4): two gamma frequency band comparison from the two studies: *SMD* = 0.104; *SE* = 0.170; 95% CI = −0.229–0.437; Z = 0.613; *P* = 0.540; *Q*-statistics = 0.161 with *P* = 0.668; *I*^2^ = 0.0%. For the comparisons of each frequency band of offline-tACS protocols, the third (different targeted brain regions) and fourth (different cognitive domains) moderator variable analyses identified no significant effects on cognition-related reaction time (Supplementary Tables 8 and 9).

## Discussion

The current systematic review and meta-analysis investigated the effects of specific tACS protocols on cognitive functions in healthy young adults. We identified 56 total studies that examined potential effects of tACS on cognitive functions using either cognitive performance or cognition-related reaction time variables. Fifty out of 56 qualified studies reported cognitive performance variable comparisons and 18 out of 56 qualified studies reported cognition-related reaction time variable comparisons. Ninety-three total comparisons from the 50 qualified studies indicated small positive overall effects on cognitive performances after active tACS protocols than sham control stimulation. Moreover, the moderator variable analyses revealed that both online- and offline-tACS protocols significantly improved cognitive performances, and further these cognitive performance improvements were observed in three specific frequency bands of tACS protocols including (a) online-tACS with theta frequency band, (b) online-tACS with gamma frequency band, and (c) offline-tACS with theta frequency band. Additional moderator analyses found that cognitive performances were improved in online-tACS with theta frequency band on PFC and PPC and online-tACS with gamma frequency band on PPC. For offline-tACS protocols, stimulation with theta frequency band on PFC significantly improved cognitive performances. Finally, online-tACS with theta frequency band significantly improved executive function and online-tACS with gamma frequency band enhanced executive function and perceptual-motor function. Offline-tACS with theta frequency band significantly improved executive function. However, we found that all specific tACS protocols failed to show any significant reduction of cognitive-related reaction time.

Our meta-analytic findings indicated that tACS protocols improved task performances in various cognitive tasks. These findings support the argument from a recent systematic review study that tACS protocols may be advantageous for improving various cognitive domains such as working memory, executive function, and declarative memory^40^. tACS protocols may induce the synchronization of neural firing timing in the cortical regions by applying low-intensity sinusoidal oscillating electrical stimulation into the scalp^106,107^. The synchronized neural firing timing in a specific brain region may contribute to improvement in cognitive functions via enhancing information-processing and memory-encoding functions^56,108,109^. However, we failed to identify a significant reduction of cognitive-related reaction time in healthy young adults. Previous studies suggested that decreased reaction time may be related to greater firing rate of cortical neurons^110,111^. In fact, greater brain activation appeared in the pre-supplementary cortex (pre-SMA)^112^, dorsolateral-prefrontal cortex (DLPFC)^48,113^, and striatum of the basal ganglia while performing faster motor actions^48,110^. For example, applying transcranial direct current stimulation (tDCS) significantly reduced reaction time during various cognitive tasks in healthy younger and older adults^114,115^ because of potential effects of tDCS on increased neural firing rate^116,117^. Potentially, given that tACS protocols may modulate the neural firing timing rather than neural firing rate in the targeted cortical regions^106^, the application of tACS may be more beneficial for improving cognitive performances (e.g., task accuracy).

The first moderator variable analysis revealed significant improvements in cognitive performances for both online- and offline-tACS conditions. Previous studies reported that applying online-tACS protocols effectively increased the synchronization between external (i.e., electrical stimulation) and internal oscillations (i.e., neural activation) in the targeted brain areas^38,40,54^. For example, when online-tACS with alpha frequency range was applied in awake non-human primates^118^ and human parieto-occipital cortex^37^, the neuron spike timing was significantly synchronized in the alpha frequency band. In addition, the neural synchronization in the occipital lobe after online-tACS protocols improved the perception of healthy younger adults^60,92^. The benefits of offline-tACS protocols on cognitive performances indicated potential after-effects that may be related to long-term potentiation (LTP) indicating increased synaptic strengthening^119–121^. Further, greater activation of N-methyl-D-aspartic (NMDA) receptors may be associated with the induction of LTP plasticity^122^. Interestingly, a prior study showed that offline-tACS protocols may cause LTP plasticity via facilitating the NMDA receptors activity in M1, because admistration of the NMDA blocker dextromethorphan diminished the effect^123^. Overall, the positive effects of both online- and offline-tACS protocols on cognitive performances support a proposition that tACS protocols may be effective for improving cognitive processing via either neural synchronization or LTP plasticity^37,124^.

Common findings from the second moderator variable analysis included that tACS protocols with theta frequency band significantly improved cognitive performances for both online and offline conditions. Further, additional moderator variable findings suggested that both online- and offline-tACS protocols with theta frequency band on either PFC or PPC enhanced cognitive performances. Specifically, we observed significant improvements in executive function after tACS protocols with theta frequency band. These findings are in line with previous findings that tACS protocols with theta frequency band was beneficial for improving various cognitive functions^40^. Specifically, tACS protocols with theta frequency band increased neural activations across the right temporal, dorsolateral-prefrontal, and frontal cortex during information encoding and retrieval processes^125,126^. Interestingly, brain oscillation patterns in frontal and posterior parietal regions were higher activated at theta frequency band when performing the cognitive tasks^52,127^, and further improvements in cognitive functions appeared with increased functional connectivity between long-distance cortical regions^128–130^. Recent functional magnetic resonance imaging studies additionally evidenced that theta-tACS protocols modulated neural connections of the hippocampal–cortical network^70,131^. These findings suggested that applying tACS protocols with theta frequency band may facilitate neural pathways within cortical regions and between cortical and sub-cortical regions contributing to improved cognitive functions.

Moreover, online-tACS protocols with gamma frequency band showed beneficial effects on cognitive performances. Interestingly, the additional moderator variable analyses demonstrated potential treatment effects of online-tACS with gamma frequency band stimulating PPC on cognitive performances, and the cognitive improvements appeared in executive function and perceptual-motor function. Previous studies suggested that rapid cortical oscillations at the gamma frequency band contributed to improved cognitive processes^132–134^. For instance, the gamma neural oscillations were observed in the medial visual cortex and anterior insula while showing better visual perception and decision-making abilities^135,136^. Moreover, applying gamma tACS protocols showed various cognitive improvements such as faster and accurate auditory and visual perceptions and memory performances^40,59,137^. Brain oscillations at the gamma frequency band may be activated via the reciprocal connection between GABAergic activity of interneurons and activity of glutamatergic pyramidal neurons^138–140^. Presumably, the gamma frequency synchronization facilitated by tACS protocols may allow precisely and flexibly transfer the neural information between the targeted brain areas^16,130,141,142^.

Although the current meta-analytic findings reveal significant positive effects of tACS protocols (i.e., theta and gamma frequency bands) on cognitive performances, the levels of cognitive improvements are relatively small (effect size range from 0.175 to 0.247). Recent findings suggested a proposition that applying tACS protocols using theta-gamma phase-amplitude coupling (PAC) can effectively modulate cognitive functions^143^. According to the cross-frequency coupling phenomenon^144,145^, cognitive function may improve when low-frequency brain oscillations reflecting information processing across largely distributed brain areas are coupled with high-frequency brain oscillations representing information processing in local brain regions^146–148^. The PAC is one of cross-frequency coupling phenomena representing that the low-frequency phase modulates the high-frequency amplitude^149–151^. Several findings posited that inducing theta-gamma PAC by delivering simultaneous theta and gamma frequency tACS over multifocal areas may facilitate neural interactions between the cortical and sub-cortical regions contributing to cognitive improvements^152–154^. In fact, applying co-stimulation protocols with theta and gamma frequency bands to the prefrontal cortex significantly improved working memory functions^143^. Moreover, theta-gamma cross-frequency coupling is important for various cognitive functions such as visual information processing and working memory^154,155^. These findings suggest that tACS protocols with co-stimulation at theta and gamma frequency bands may be a viable option to increase cognitive improvements by inducing theta-gamma PAC that potentially reinforces neural communications across brain regions^138^.

Despite quantitative findings indicating potential effective tACS protocols for cognitive functions in healthy younger adults, these are some limitations. First, given that the current meta-analysis focused on altered cognitive functions in healthy younger adults, the relatively small effects of tACS protocols may be influenced by a ceiling effect^55^. Thus, future studies need to quantity beneficial effects of tACS protocols on cognitive functions for participants with cognitive impairments (e.g., older adults and patients with neurological diseases). Second, 20 studies in this meta-analysis reported potential side effects after tACS protocols. Tingling and itching are frequently observed after transcranial electrical stimulation^156^. In particular, tACS may cause phosphenes in which artificial light flashing or shimmering affects visual perceptions and concentration. Phosphenes often appeared when either tACS applied adjacent to occipital cortex or the stimulation intensity greater than 1.5 mA provided^157^. To minimize these potential side effects, providing individualized current intensity thresholds and electrode montage positions should be considered in future studies.

The current systematic review and meta-analysis revealed that applying tACS protocols significantly improved cognitive performances in healthy younger adults. Moreover, moderator variable analyses found the positive effects on cognitive performances for both online- and offline-tACS conditions. Specifically, significant improved cognitive performances after tACS protocols were observed in following frequency bands: (a) online-tACS with theta frequency band, (b) online-tACS with gamma frequency band, and (c) offline-tACS with theta frequency band. Further, cognitive performances were improved in online- and offline-tACS with theta frequency band on either PFC or PPC, and further both online- and offline-tACS with theta frequency band enhanced executive function. Online-tACS with gamma frequency band on PPC was effective for improving cognitive performances, and the cognitive improvements appeared in executive function and perceptual-motor function. These meta-analytic findings suggest that applying specific tACS protocols can facilitate improvements in various cognitive performances for healthy young adults. Importantly, previous studies revealed that the changes in PAC characteristics caused by decreased theta frequency band may be related to cognitive impairments in older adults as well as patients with neurologic diseases such as schizophrenia, Alzheimer’s disease, and epilepsy^150,158,159^. Thus, future studies should investigate whether tACS protocols with co-stimulation at theta and gamma frequency bands are beneficial for improving cognitive functions in older adults and patients with neurological diseases.

## Methods

### Literature search and study inclusion

Based on the Preferred Reporting Items for Systematic Reviews and Meta-Analysis (PRISMA) statement^160^, we conducted a systematic review and meta-analysis. The computerized literature search from November 15, 2020 to October 29, 2021 identified potential studies via PubMed and Web of Science. We used the following keywords: (tACS or transcranial alternating current stimulation) and (reaction time or response time or RT or cognitive or cognition or cognitive performance or cognitive function) and (healthy and adults). The inclusion criteria for this meta-analysis were: (a) studies recruiting cognitively healthy young adults, (b) studies performed quantitative evaluation on either cognitive performance or cognition-related reaction time, (c) studies included sham stimulation controls, and (d) studies with a randomized control trial or crossover design. We excluded review articles, case studies, animal studies, and articles that were not related to our main topic (e.g., elderly population, participants with specific disorder, and no tACS effects reported).

### Cognitive function outcome measures

To investigate changes in cognitive function after tACS protocols, we focused on two primary outcome measures including (a) cognitive performance variable (i.e., accuracy, precision, correct response, error rated, score, and hit rated) and (b) cognition-related reaction time variable (i.e., time interval between stimuli and the completion of the cognitive task). To examine the effects of tACS on specific cognitive domains, we categorized cognitive functions into five components^51,114,161^: (a) perceptual-motor function (e.g., visual perception and perceptual-motor integration), (b) learning and memory (e.g., free recall, recognition, long-term memory, and implicit learning), (c) executive function / complex attention (e.g., planning, decision-making, working memory, selective attention, and inhibition), (d) language (e.g., object naming, fluency, and receptive language), and (e) social cognition (e.g., recognition of emotions, theory of mind, and insight).

### Meta-analytic approaches for data synthesis

Using the meta-analysis software (Comprehensive Meta-Analysis software ver. 3.2, Englewood, NJ, USA), we performed all meta-analysis procedures. The effect sizes for the parallel group studies were quantified by the difference in task performance and reaction time between the active tACS and sham control groups at the post-test using standardized mean difference (SMD) with a 95% confidence interval (CI)^162^. Consistent with previous suggestions^162–165^, we used a paired analysis for crossover studies to calculate the SMD (e.g., values of sample size and mean difference with *P*-values or sample size and mean difference with standard error). This approach may correctly report clinically important heterogeneity in the meta-analysis while including crossover trials into a meta-analysis^114,165^. More positive values of SMD denoted greater positive effects on cognitive functions after active tACS than the sham control stimulation. Finally, all effect size calculations were based on the random-effects meta-analysis models because of the conventional assumption that individual studies have various experiment characteristics (e.g., participants and experimental protocols)^166^.

To estimate the heterogeneity levels across multiple comparisons, we conducted the Higgins and Green *I*^2^ test that demonstrates the percentage of heterogeneity between 0 to 100%^167^. The heterogeneity levels with 25, 50, and 75% of *I*^2^ indicate low, moderate, and high variability across studies, respectively^168^. In addition, we used Cochran’s *Q* and *P*-value, the heterogeneity significance test based on the chi-squared distribution. A *P*-value less than 0.05 for the *Q*-statistic indicates significant levels of heterogeneity between studies^162^. To quantify potential publication bias, we applied two methods. First, an original funnel plot and a revised funnel plot after the trim and fill technique were compared as a visual estimation of the changes in the overall effect sizes^169^. When no values overlapped between the original overall effect size and corrected overall effect size, a significant publication bias may exist. Second, we conducted Egger’s regression test providing the degree of asymmetry for the funnel plot by quantifying the intercept in the regression of standard normal deviates against precision^170^. The *P*-value for the intercept (*β*0) less than 0.05 implicates a significant publication bias across the comparisons.

To specify the effects of various tACS protocols on cognitive function, we performed moderator variable analyses. The first moderator variable analysis estimated different timing of tACS protocols: (a) online-tACS (i.e., applied tACS protocols during cognitive tasks) and (b) offline-tACS (i.e., using tACS protocols before executing cognitive tasks). In the second moderator variable analysis, we determined whether the effect sizes of specific frequency bands for tACS protocols were different: (a) delta band (1–3 Hz), (b) theta band (4–7 Hz), (c) alpha band (8–12 Hz), (d) beta band (13–30 Hz), (e) gamma band (31–139 Hz), and (f) ripple band (140 Hz). The third moderator variable analysis examined the potential effects of targeted brain regions for tACS protocols on cognitive functions: (a) PFC, (b) PPC, (c) TC, and (d) Multi. The fourth moderator variable analysis investigated the effects of tACS protocols on different cognitive domains: (a) perceptual-motor function, (b) learning and memory (c) executive function / complex attention, (d) language, and (e) social cognition.

### Methodological quality assessment

Two authors (TLL and HAL) independently conducted the methodological quality of the included studies in the current meta-analysis using version 2 of a revised Cochrane risk of bias tool^171^. The assessment tool consists of six questionnaire domains: (a) randomization process, (b) timing of identification or recruitment of participants, (c) deviations from intended interventions, (d) missing outcome data, (e) measurement of the outcome, and (f) selection of the reported result. The methodological quality questionnaire can be evaluated on three levels: (a) low risk of bias, (b) high-risk bias, and (c) some concern.

### Reporting summary

Further information on research design is available in the Nature Research Reporting Summary linked to this article.

## Supplementary information

Supplementary Material

Reporting Summary

## Data Availability

All data generated or analyzed during this study are included in this manuscript.
