# Supplementary material for: A meta-analysis showing improved cognitive performance in healthy young adults with transcranial alternating current stimulation
Source: NPJ Sci Learn. 2023 Jan 3;8:1. doi: 10.1038/s41539-022-00152-9 (PMC9807644; doi:10.1038/s41539-022-00152-9)
Supplement: Supplementary file 1 — Supplementary Material [file 41539_2022_152_MOESM1_ESM.pdf]

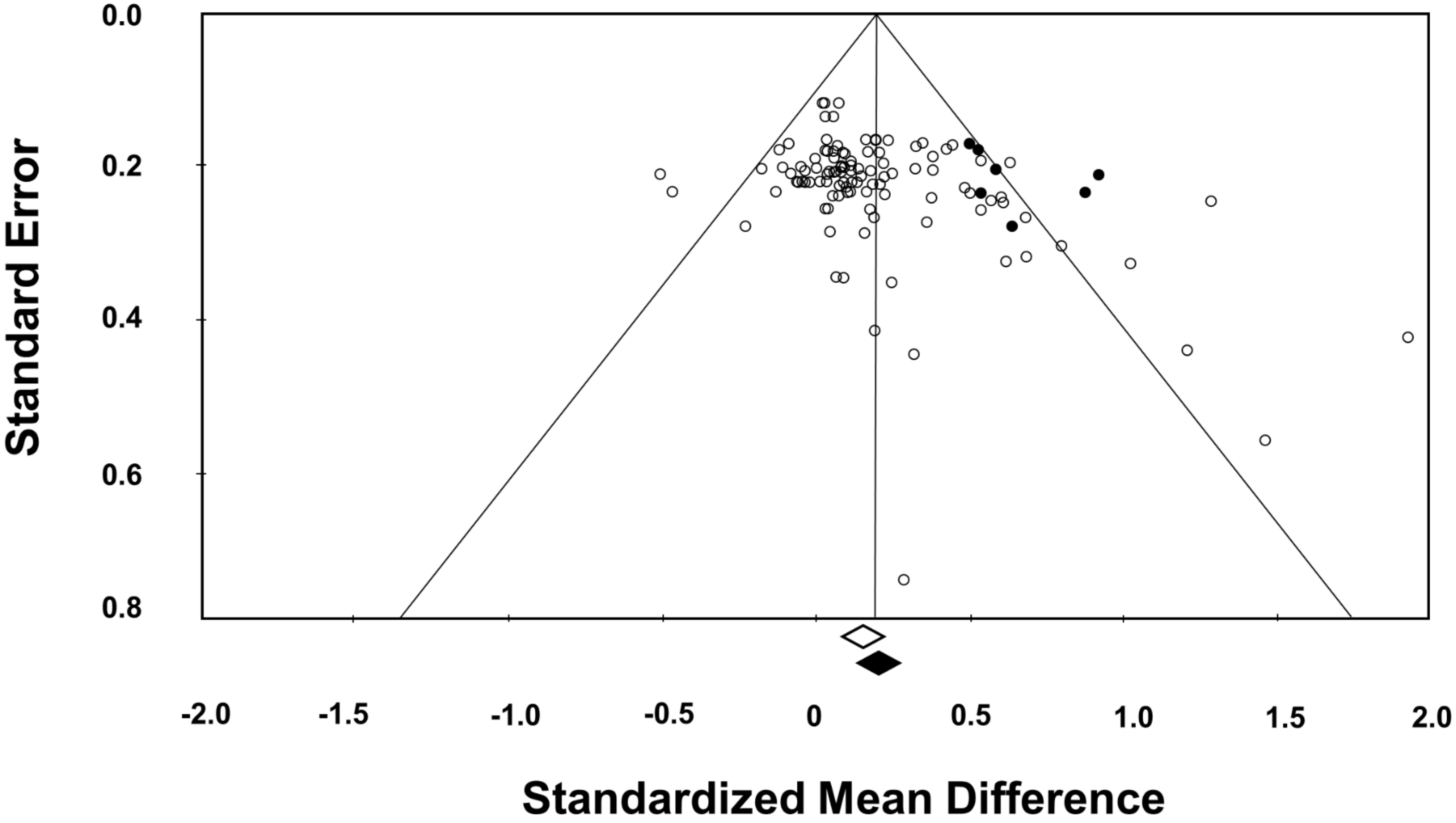

Supplementary Figure 1. A revised funnel plot for the cognitive performance comparisons.

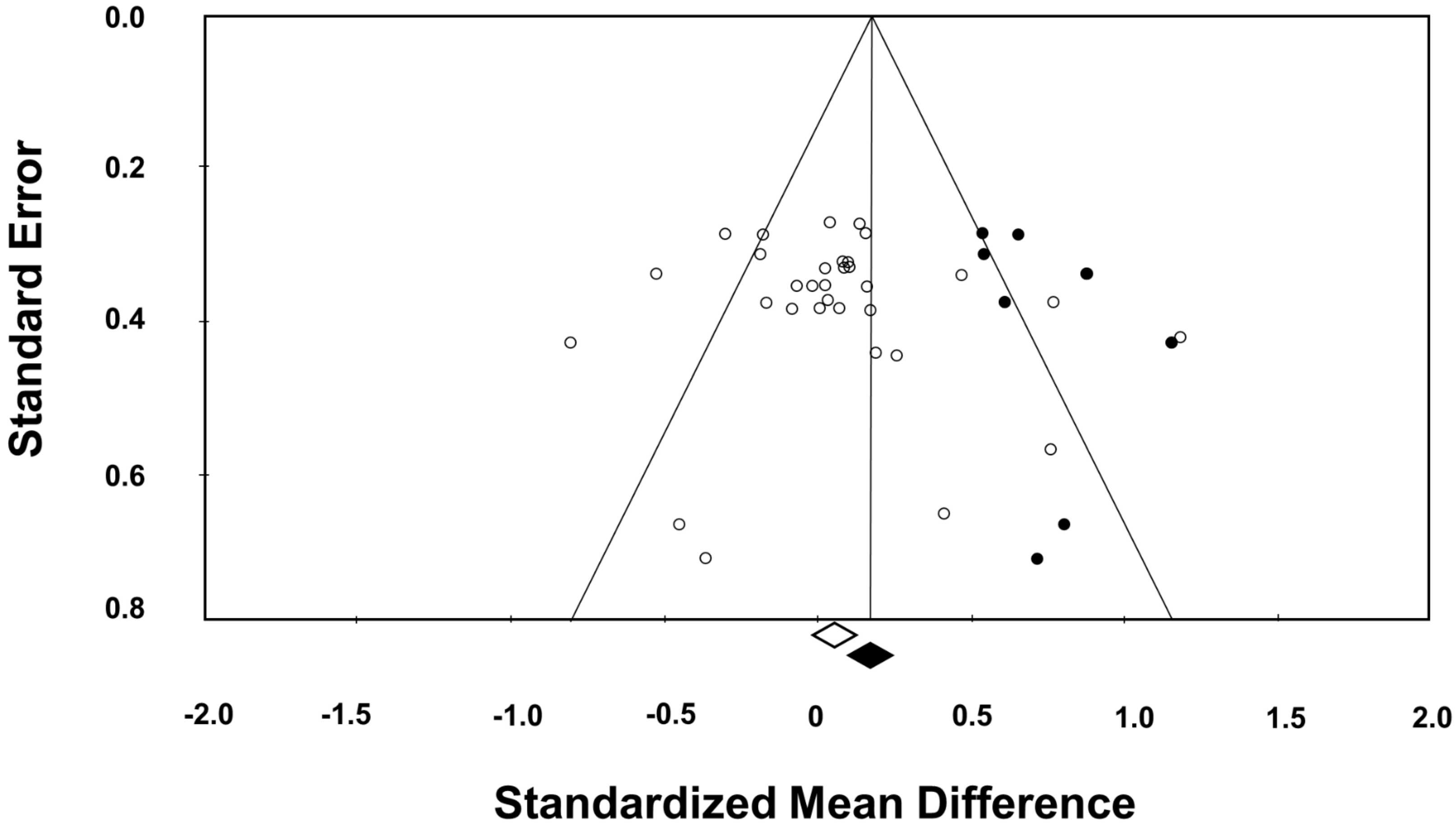

Supplementary Figure 2. A revised funnel plot for the cognition-related reaction time comparisons.

Study Name	Outcome measure	SMD	LL	UL	*P*-value
Giustiniani 2019	Serial reaction time task (Learning and memory); tACS (δ) vs. Sham	-0.08	-0.56	0.39	0.735
	**Online-delta frequency band overall**	**-0.08**	**-0.56**	**0.39**	**0.735**
Alekseichuk 2017	Two-back visual spatial task (Executive function / Complex attention); tACS (θ) vs. Sham	-0.52	-0.94	-0.11	0.014
Brauer 2018	Go/Nogo task (Executive function / Complex attention); tACS (θ) vs. Sham	0.77	0.31	1.24	0.001
Meiron 2014	N-back task (Executive function/Complex attention); tACS (θ) vs. Sham	0.42	-0.39	1.23	0.312
Polanía 2012	Delayed letter discrimination task (Executive function / Complex attention); tACS (θ) vs. Sham	-0.80	-1.34	-0.27	0.003
Santarnecchi 2013	Fluid intelligence task (Executive function / Complex attention); tACS (θ) vs. Sham	-0.06	-0.50	0.38	0.776
Santarnecchi 2016	Abstract-reasoning task / Change-localization (Executive function / Complex attention); tACS (θ) vs. Sham	0.10	-0.30	0.51	0.611
Vosskuhl 2015	Digit span task/N-back task (Executive function / Complex attention); tACS (θ) vs. Sham	0.77	0.06	1.47	0.033
Zavecz 2020	Alternating serial reaction time task (Learning and memory); tACS (θ) vs. Sham	-0.19	-0.57	0.20	0.349
	**Online-theta frequency band overall**	**0.03**	**-0.34**	**0.40**	**0.880**
Hopfinger 2017	Visual attention task (Executive function / Complex attention); tACS (α) vs. Sham	0.09	-0.32	0.50	0.669
Kasten 2018	Mental rotation task (Perceptual-motor function); tACS (α) vs. Sham	-0.36	-1.25	0.52	0.422
Pollik 2015	Serial reaction time task (Learning and memory); tACS (α) vs. Sham	0.26	-0.29	0.81	0.356
Santarnecchi 2013	Fluid intelligence task (Executive function / Complex attention); tACS (α) vs. Sham	-0.12	-0.31	0.57	0.955
Santarnecchi 2019	Insight task (Executive function / Complex attention); tACS (α) vs. Sham	-0.18	-0.53	0.18	0.324
Schuhmann 2019	Endogenous attention task (Perceptual-motor function); tACS (α) vs. Sham	0.14	-0.21	0.48	0.417
	**Online-alpha frequency band overall**	**0.02**	**-0.16**	**0.20**	**0.82**
Pollik 2015	Serial reaction time task (Learning and memory); tACS (β) vs. Sham	0.19	-0.36	0.74	0.493
Santarnecchi 2013	Fluid intelligence task (Executive function / Complex attention); tACS (β) vs. Sham	0.16	-0.40	0.48	0.468
	**Online-beta frequency band overall**	**0.18**	**-0.17**	**0.52**	**0.320**
Giustiniani 2019	Serial reaction time task (Learning and memory); tACS (γ) vs. Sham	0.01	-0.46	0.49	0.964
Hopfinger 2017	Visual attention task (Executive function / Complex attention); tACS (γ) vs. Sham	0.11	-0.30	0.52	0.610
Loffler 2018	Visual two-choice task (Perceptual-motor function); tACS (γ) vs. Sham	-0.45	-1.28	0.38	0.287
Polanía 2012	Delayed letter discrimination task (Executive function / Complex attention); tACS (γ) vs. Sham	-0.17	-0.63	0.30	0.479
Polanía 2015	Decision-making task (Executive function / Complex attention); tACS (γ) vs. Sham	-0.30	-0.66	0.05	0.095
Santarnecchi 2013	Fluid intelligence task (Executive function / Complex attention); tACS (γ) vs. Sham	0.03	-0.41	0.46	0.910
Santarnecchi 2016	Abstract-reasoning task / Change-localization (Executive function / Complex attention); tACS (γ) vs. Sham	1.19	0.67	1.72	<0.001
Santarnecchi 2016	Abstract-reasoning task / Change-localization (Executive function / Complex attention); tACS (γ) vs. Sham	0.04	-0.30	0.38	0.810
Santarnecchi 2019	Insight task (Executive function / Complex attention); tACS (γ) vs. Sham	0.16	-0.20	0.51	0.380
	**Online-gamma frequency band overall**	**0.08**	**-0.18**	**0.33**	**0.557**

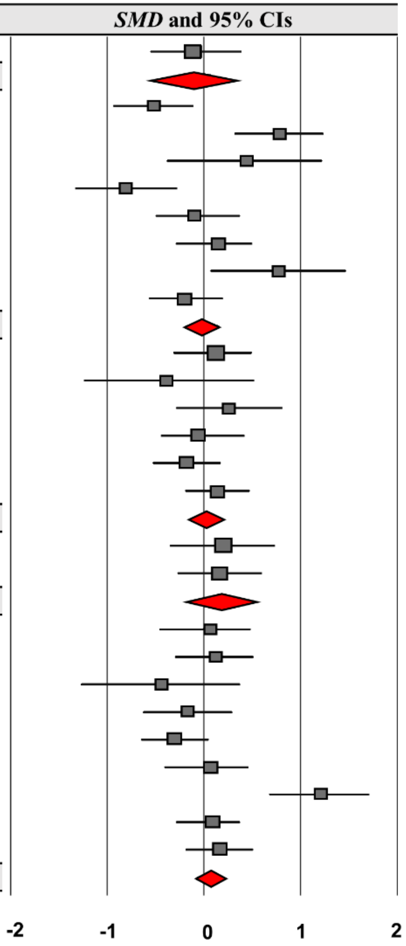

Abbreviations. CIs: confidence intervals; LL: lower limit; SMD: standardized mean difference; UL: upper limit; δ: delta bend (1-3 Hz); θ: theta band (4-8 Hz); α: alpha band (9-13 Hz); β: beta band (14-30 Hz); γ: gamma band (30-139 Hz).

Note. Red diamonds indicate an overall effects size. More positive values indicate greater improvement in cognitive performance.

Supplementary Figure 3. Meta-analytic findings for the cognition-related reaction time on online-tACS protocols with specific frequency band comparisons.

Study Name	Outcome measure	SMD	LL	UL	*P*-value	***SMD* and 95% CIs**
Giustiniani 2019	Serial reaction time task (Learning and memory); tACS (δ) vs. Sham	0.07	-0.41	0.55	0.769	
	**Offline-delta frequency band overall**	**0.07**	**-0.41**	**0.55**	**0.769**	
Brauer 2018	Go/Nogo task (Executive function / Complex attention); tACS (θ) vs. Sham	0.03	-0.38	0.44	0.895	
	**Offline-theta frequency band overall**	**0.03**	**-0.38**	**0.44**	**0.895**	
Moliadze 2019	Phonological decision task (Executive function / Complex attention); tACS (α) vs. Sham	0.48	0.05	0.90	0.027	
	**Offline-alpha frequency band overall**	**0.48**	**0.05**	**0.90**	**0.027**	
Moliadze 2019	Phonological decision task (Executive function / Complex attention); tACS (β) vs. Sham	0.09	-0.31	0.49	0.667	
	**Offline-beta frequency band overall**	**0.09**	**-0.31**	**0.49**	**0.667**	
Giustiniani 2019	Serial reaction time task (Learning and memory); tACS (γ) vs. Sham	0.18	-0.30	0.65	0.475	
Hoy 2015	N-back task (Executive function / Complex attention); tACS (γ) vs. Sham	0.04	-0.42	0.50	0.871	
	**Offline-gamma frequency band overall**	**0.10**	**-0.23**	**0.44**	**0.540**	

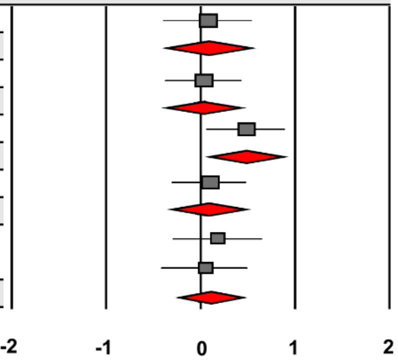

Abbreviations. CIs: confidence intervals; LL: lower limit; SMD: standardized mean difference; UL: upper limit; δ: delta bend (1-3 Hz); θ: theta band (4-8 Hz); α: alpha band (9-13 Hz); β: beta band (14-30 Hz); γ: gamma band (30-139 Hz).

Note. Red diamonds indicate an overall effects size. More positive values indicate greater improvement in cognitive performance.

Supplementary Figure 4. Meta-analytic findings for the cognition-related reaction time on offline-tACS protocols with specific frequency band comparison

Supplementary Table 1. Side effect information for participants.

Study	Presence of Side Effect	Total # of Participant	# of Participants with Side effects	Specific Side effects
Alekseichuk (2017) [52]	Not reported	25	–	–
Alekseichuk (2020) [84]	No	25	–	–
Ambrus (2015) [53]	Not reported	18	–	–
Antonenko (2016) [54]	Yes	12	12	Pain, tingling, itching, burning, tiredness, and loss of concentration
Brauer (2018) [55]	Yes	23	Exact number is not available	Tingling, itching, flickering, burning, pain, headache, fatigue, and loss of concentration
Braun (2017) [85]	Yes	Exp 1) 36<br>Exp 2) 36	36	Phosphenes
Brignani (2013) [56]	Yes	96	61	Itching and pinch
Deng (2019) [86]	Not reported	Exp 1) 20<br>Exp 2) 18	–	–
Feurra (2016) [45]	No	14	–	–
Fusco (2018) [57]	Yes	36	Exact number is not available	Itching, tingling, prickling, heat, burning, and flickering
Giustiniani (2019) [58]	No	17	–	–
Grabner (2018) [87]	Yes	22	22	Discomfort
Gutteling (2017) [88]	Yes	22	Exact number is not available	Tingling*
Hopfinger (2017) [89]	No	23	–	–
Hoy (2015) [59]	Not reported	18	–	–
Janik (2015) [60]	Not reported	22	–	–
Jaušovec (2014, BP) [61]	Not reported	24	–	–
Jaušovec (2014, AP) [62]	Not reported	36	–	–
Javadi (2017) [90]	Not reported	17	–	–
Kasten (2018) [91]	Not reported	20	–	–
Laczó (2012) [92]	Yes	20	2	Headache
Lang (2019) [63]	Yes	37	Exact number is not available	Tingling, burning, and discomfort
Loffler (2018) [64]	Yes	23	23	tiring and difficulty to concentration
Luft (2018) [65]	Not reported	Exp 1) 29<br>Exp 2) 36	–	–
Lustenberger (2015) [66]	Yes	Exp 1) 19<br>Exp 2) 20	39	Flickering
Marchesotti (2020) [67]	Yes	15	Exact number is not available	Pain, warming, tingling and anxiety, fatigue, and difficulty to concentration
Meier (2019) [68]	No	26	–	–
Meiron (2014) [69]	No	24	–	–
Meng (2021) [70]	Yes	18	1	Discomfort

Abbreviations. AP: published in the Acta Psychologica; BP: published in the Biological Psychology; Exp: experiment. Note. Asterisk (*) indicates that side effects appeared at the initial stimulation and disappeared during experiments.

Supplementary Table 1. Side effect information for participants (Continued).

Study	Presence of Side Effect	Total # of Participant	# of Participants with Side effects	Specific Side effects
Moliadze (2019) [93]	Yes	24	24	Tiredness, itching, flickering, concentration problems, headache, and shock-like sensations
Neubauer (2017) [94]	Not reported	20	–	Not reported
Nomura (2019) [71]	No	36	–	–
Pahor (2014) [72]	Not reported	28	–	–
Pahor (2016) [95]	Not reported	18	–	–
Polanía (2012) [73]	Yes	36	36	Itching*
Polanía (2015) [74]	Not reported	86	–	–
Pollok (2015) [75]	Yes	13	11	Itching, tingling, and flickering
Reinhart (2017) [96]	Not reported	Exp 1) 30<br>Exp 2) 30<br>Exp 3) 30	–	–
Riecke (2015) [97]	Not reported	20	–	–
Riecke (2018) [98]	Not reported	20	–	–
Santarnecchi (2013) [76]	Yes	20	4	Tingling* and flickering
Santarnecchi (2016) [77]	Yes	Exp 1) 24<br>Exp 2) 34	2	Tingling* and flickering
Santarnecchi (2019) [78]	Not reported	31	–	–
Schuhmann (2019) [79]	Not reported	34	–	–
Sela (2012) [80]	Yes	27	Exact number is not available	Itching*
Strüber (2014) [99]	No	Exp 1) 17<br>Exp 2) 13<br>Exp 3) 15	–	–
Tseng (2016) [100]	No	Exp 1) 20<br>Exp 2) 20	–	–
Tseng (2018) [101]	No	Exp 1) 24<br>Exp 2) 24	–	–
Violante (2017) [81]	No	10	–	–
Vosskuhl (2015) [46]	Not reported	33	–	–
Wischnewski (2016) [82]	Yes	50	7	Itching*
Wöstmann (2018) [102]	Yes	20	20	Tingling, difficulty in concentration, and tiredness
Wynn (2020) [83]	Yes	54	13	Tingling, itching, dizziness, and headache
Zavecz (2020) [103]	Not reported	26	–	–
Zoefel (2018) [104]	Yes	17	Exact number is not available	Not reported
Zoefel (2020) [105]	Not reported	Exp 1) 27<br>Exp 2) 19	–	–

Abbreviations. AP: published in the Acta Psychologica; BP: published in the Biological Psychology; Exp: experiment. Note. Asterisk (*) indicates that side effects appeared at the initial stimulation and disappeared during experiments.

Supplementary Table 2. Online-tACS and cognitive performances for different targeted brain regions.

Comparison Name	Comparison Number	SMD	SE	LL	UL	*Z*-score	*P*-values
Delta frequency band							
PFC	1	0.447	0.175	0.104	0.790	2.557	0.011
TC	3	0.061	0.118	-0.170	0.292	0.516	0.606
Theta frequency band							
TC	2	0.102	0.159	-0.209	0.414	0.644	0.519
Multi	2	-0.140	0.364	-0.854	0.575	-0.383	0.702
Alpha frequency band							
PFC	5	0.013	0.092	-0.048	0.314	1.443	0.149
PPC	7	-0.037	0.089	-0.212	0.138	-0.415	0.678
TC	2	0.093	0.146	-0.193	0.380	0.638	0.523
Multi	1	-0.040	0.224	-0.478	0.399	-0.178	0.859
Beta frequency band							
PFC	3	0.107	0.105	-0.098	0.312	1.024	0.306
PPC	2	0.459	0.538	-0.595	1.514	0.854	0.393
Gamma frequency band							
PFC	10	0.108	0.066	-0.022	0.237	1.633	0.102
TC	1	0.636	0.197	0.250	1.022	3.229	0.001
Multi	5	0.184	0.094	0.000	0.367	1.963	0.050

Abbreviations. LL: lower limb; Multi: multiple regions; PFC: prefrontal cortex; PPC: posterior parietal cortex; SE: standard error; SMD: standardized mean difference, TC: temporal cortex; UL: upper limb.

Supplementary Table 3. Online-tACS and cognitive performances for different cognitive domains.

**Comparison Name**	**Comparison Number**	**SMD**	**SE**	**LL**	**UL**	**Z-score**	***P*-values**
Delta frequency band							
Executive function / complex attention	4	0.178	0.106	-0.030	0.387	1.675	0.094
Theta frequency band							
Perceptual-motor function	5	0.048	0.105	-0.157	0.254	0.463	0.644
Learning and memory	4	0.202	0.170	-0.007	0.411	1.892	0.059
Alpha frequency band							
Perceptual-motor function	3	-0.032	0.141	-0.308	0.243	-0.229	0.819
Learning and memory	1	0.059	0.136	-0.208	0.326	0.436	0.663
Executive function / complex attention	11	0.058	0.069	-0.078	0.193	0.837	0.403
Beta frequency band							
Perceptual-motor function	1	-0.048	0.204	-0.448	0.352	-0.236	0.814
Executive function / complex attention	4	0.255	0.165	-0.068	0.579	1.546	0.122

Abbreviations. LL: lower limb; SE: standard error; SMD: standardized mean difference, UL: upper limb.

Supplementary Table 4. Offline-tACS and cognitive performances for different targeted brain regions.

**Comparison Name**	**Comparison Number**	**SMD**	**SE**	**LL**	**UL**	***Z*-score**	***P*-values**
Theta frequency band							
PPC	6	0.179	0.106	-0.029	0.387	1.689	0.091
TC	1	0.572	0.247	0.087	1.057	2.310	0.021
Alpha frequency band							
PFC	2	0.131	0.155	-0.173	0.435	0.845	0.398
PPC	1	-0.055	0.224	-0.493	0.384	-0.244	0.807
TC	1	-0.047	0.236	-0.509	0.415	-0.200	0.842
Beta frequency band							
PPC	1	0.077	0.204	-0.324	0.478	0.377	0.706
Gamma frequency band							
PFC	3	0.066	0.143	-0.213	0.345	0.463	0.644
TC	1	0.041	0.258	-0.465	0.547	0.158	0.874

Abbreviations. LL: lower limb; PFC: prefrontal cortex; PPC: posterior parietal cortex; SE: standard error; SMD: standardized mean difference, TC: temporal cortex; UL: upper limb.

Supplementary Table 5. Offline-tACS and cognitive performances for different targeted brain regions.

Comparison Name	Comparison Number	SMD	SE	LL	UL	Z-score	*P*-values
Theta frequency band							
Perceptual-motor function	2	0.335	0.231	-0.119	0.788	1.446	0.148
Learning and memory	1	0.162	0.291	-0.408	0.731	0.557	0.577
Alpha frequency band							
Executive function / complex attention	4	0.044	0.112	-0.175	0.264	0.394	0.693
Beta frequency band							
Executive function / complex attention	1	0.077	0.204	-0.324	0.478	0.377	0.706
Gamma frequency band							
Learning and memory	1	0.288	0.747	-1.176	1.751	0.385	0.700
Executive function / complex attention	3	0.054	0.127	-0.195	0.302	0.423	0.672

Abbreviations. LL: lower limb; SE: standard error; SMD: standardized mean difference, UL: upper limb.

Supplementary Table 6. Online-tACS and cognition-related reaction time for different targeted brain regions.

Comparison Name	Comparison Number	SMD	SE	LL	UL	Z-score	*P*-values
Delta frequency band							
PFC	1	-0.082	0.243	-0.558	0.394	-0.338	0.735
Theta frequency band							
PPC	4	0.285	0.201	-0.109	0.679	1.418	0.156
Multi	4	-0.233	0.268	-0.758	0.292	-0.869	0.385
Alpha frequency band							
PFC	2	0.093	0.175	-0.251	0.436	0.530	0.596
PPC	4	-0.005	0.104	-0.210	0.199	-0.05	0.960
Beta frequency band							
PPC	2	0.175	0.175	-0.169	0.518	0.995	0.320
Gamma frequency band		0.297	0.256	-0.204	0.799	1.162	0.245
PFC	4	0.297	0.256	-0.204	0.799	1.162	0.245
PPC	2	-0.050	0.250	-0.541	0.440	-0.201	0.841
TC	1	0.159	0.181	-0.196	0.513	0.878	0.380
Multi	2	-0.253	0.144	-0.534	0.029	-1.757	0.079

Abbreviations. LL: lower limb; Multi: multiple regions; PFC: prefrontal cortex; PPC: posterior parietal cortex; SE: standard error; SMD: standardized mean difference, TC: temporal cortex; UL: upper limb.

Supplementary Table 7. Online-tACS and cognition-related reaction time for different cognitive domains.

Comparison Name	Comparison Number	SMD	SE	LL	UL	Z-score	*P*-values
Delta frequency band							
Learning and memory	1	-0.082	0.243	-0.558	0.394	-0.338	0.735
Theta frequency band							
Learning and memory	1	-0.186	0.198	-0.574	0.201	-0.943	0.346
Executive function / complex attention	7	0.068	0.224	-0.370	0.507	0.305	0.760
Alpha frequency band		0.062	0.182	-0.295	0.419	0.339	0.735
Perceptual-motor function	2	0.062	0.182	-0.295	0.419	0.339	0.735
Learning and memory	1	0.260	0.282	-0.292	0.813	0.923	0.356
Executive function / complex attention	3	-0.050	0.117	-0.279	0.179	-0.427	0.669
Beta frequency band							
Learning and memory	1	0.192	0.280	-0.357	0.740	0.685	0.493
Executive function / complex attention	1	0.163	0.225	-0.278	0.605	0.726	0.468
Gamma frequency band		-0.450	0.423	-1.278	0.379	-1.064	0.287
Perceptual-motor function	1	-0.450	0.423	-1.278	0.379	-1.064	0.287
Learning and memory	1	0.011	0.243	-0.464	0.486	0.045	0.964
Executive function / complex attention	7	0.127	0.153	-0.173	0.427	0.829	0.407

Abbreviations. LL: lower limb; SE: standard error; SMD: standardized mean difference, UL: upper limb.

Supplementary Table 8. Offline-tACS and cognition-related reaction time for different brain regions.

Comparison Name	Comparison Number	SMD	SE	LL	UL	*Z*-score	*P*-values
Delta frequency band							
PFC	1	0.071	0.243	-0.405	0.547	0.294	0.769
Theta frequency band							
PFC	1	0.028	0.209	-0.381	0.436	0.133	0.895
Alpha frequency band							
PFC	1	0.475	0.215	0.053	0.897	2.208	0.027
Beta frequency band							
PFC	1	0.088	0.205	-0.313	0.489	0.431	0.667
Gamma frequency band							
PFC	2	0.104	0.170	-0.229	0.437	0.613	0.540

Abbreviations. LL: lower limb; PFC: prefrontal cortex; PPC: posterior parietal cortex; SE: standard error; SMD: standardized mean difference, UL: upper limb.

Supplementary Table 9. Offline-tACS and cognition-related reaction time for different cognitive domains.

Comparison Name	Comparison Number	SMD	SE	LL	UL	Z-score	*P*-values
Delta frequency band							
Learning and memory	1	0.071	0.243	-0.405	0.547	0.294	0.769
Theta frequency band							
Executive function / complex attention	1	0.028	0.209	-0.381	0.436	0.133	0.895
Alpha frequency band							
Executive function / complex attention	1	0.475	0.215	0.053	0.897	2.208	0.027
Beta frequency band							
Executive function / complex attention	1	0.088	0.205	-0.313	0.489	0.431	0.667
Gamma frequency band							
Learning and memory	1	0.175	0.244	-0.304	0.653	0.714	0.475
Executive function / complex attention	1	0.038	0.236	-0.424	0.500	0.162	0.871

Abbreviations. LL: lower limb; SE: standard error; SMD: standardized mean difference, UL: upper limb.
